# Nematicidal glycosylated resorcylic acid lactones from the fungus *Pochonia chlamydosporia* PC-170 and their key biosynthetic genes

**DOI:** 10.3389/fmicb.2024.1385255

**Published:** 2024-04-04

**Authors:** Zeyu Li, Ning Luo, Wenwen Zhang, Raja Asad Ali Khan, Jian Ling, Jianlong Zhao, Yuhong Yang, Zhenchuan Mao, Bingyan Xie, Ligang Zhou, Yan Li

**Affiliations:** ^1^State Key Laboratory of Vegetable Biobreeding, Institute of Vegetables and Flowers, Chinese Academy of Agricultural Sciences, Beijing, China; ^2^Department of Plant Pathology, College of Plant Protection, China Agricultural University, Beijing, China; ^3^Biocontrol Engineering Laboratory of Crop Diseases and Pests of Gansu Province, College of Plant Protection, Gansu Agricultural University, Lanzhou, China

**Keywords:** *Pochonia chlamydosporia*, RALs, nematicidal activity, *Meloidogyne incognita*, biosynthetic gene

## Abstract

Chemical study of the nematicidal biocontrol fungus *Pochonia chlamydosporia* PC-170 led to discovery of six resorcylic acid lactones (RALs), including three nematicidal glycosylated RALs, monocillin VI glycoside **(1)**, colletogloeolactone A **(2)** and monocillin II glycoside **(3)**, and three antibacterial non-glycosylated RALs, monocillin VI **(4)**, monocillin IV **(5)** and monocillin II **(6)**. The planar structure of the new compound monocillin VI glycoside **(1)** was elucidated using HRESIMS and NMR data, and its monosaccharide configuration was further determined through sugar hydrolysis experiment and GC–MS analysis method. Furthermore, their two biosynthetic-related PKS genes, *pchE* and *pchI*, were identified through the gene knockout experiment. The glycosylated RALs **1–3** exhibited nematicidal activity against *Meloidogyne incognita*, with LC_50_ values of 94, 152 and 64 *μ*g/mL, respectively, and thus had great potential in the development of new nematicidal natural products to control *M. incognita* in the future.

## 1 Introduction

*Meloidogyne incognita*, a phytopathogenic nematode, adversely influences crop growth via its parasitic activity predominantly within the root system of plants (Li et al., 2015). The manifestations of this infestation may include plant stunting, leaf yellowing, and other associated symptoms, with severe instances culminating in plant mortality (Jones et al., 2013; Goverse and Smant, 2014). Simultaneously, the injury caused by the nematode is thought to create a favorable environment for the proliferation of other phytopathogens. It is crucial to implement preventive or restrictive measures to control the spread of this pest. Currently, chemical control methods are predominantly utilized to manage *M. incognita*, emphasizing the urgent need to explore natural products exhibiting nematicidal properties.

*Pochonia chlamydosporia*, a facultative parasitic fungus, manifests a preference for parasitizing root-knot nematode egg masses and females while also displaying significant potential as a biocontrol agent against *M. incognita* (Khan et al., 2021; Bo et al., 2022), attracting considerable research attention (Manzanilla-López et al., 2013; Zhuang et al., 2021). It was reported that the main secondary metabolites produced by *P. chlamydosporia* mainly belong to resorcylic acid lactones (RALs), which exhibited various biological activities, including antiviral (Hellwig et al., 2003), antiparasitic (Hellwig et al., 2003), cytotoxic (Sugawara et al., 1992), antimalarial (Isaka et al., 2002), antifungal (Xu et al., 2012) and antibacterial (Qin et al., 2019). We initially investigated a strain of *P. chlamydosporia* PC-170, and identified seven RALs with modest antibacterial activities against the plant pathogen *Xanthomonas campestris* (Qin et al., 2019). However, after nematicidal activity evaluation, none of these RALs showed efficacy against *M. incognita*. Since the crude extract of the fermentation cultures of *P. chlamydosporia* PC-170 still showed strong inhibitory activity against *M. incognita*, we continued screening for the potent nematicidal natural products derived from the crude extract of PC-170. Bioassay-guided fractionation of this extract led to the identification of six RALs compounds, including a new RAL, monocillin VI glycoside (**1**), together with five known analogs, colletogloeolactone A (**2**), monocillin II glycoside (**3**), monocillin VI (**4**), monocillin IV (**5**) and monocillin II (**6**). Three glycosides (**1**–**3**) exhibited noteworthy nematicidal activity with inhibitory activity against *M. incognita* with LC_50_ values of 94, 152 and 64 *μ*g/mL, respectively. In this paper, we report the isolation and structure elucidation of these RALs and their nematicidal activities. Moreover, we also confirmed the functions of two key radicicol biosynthetic genes (*pchE* and *pchI*) in the *P. chlamydosporia* strain PC-170. This study contributes valuable insights into the management of *M. incognita* and serves as a fundamental basis for developing nematicidal natural products.

## 2 Materials and methods

### 2.1 Materials

#### 2.1.1 Experimental procedures

Semi-preparative high-performance liquid chromatography (HPLC) was performed on an Agilent 1,260 G7111A Quaternary Pump equipped with a G7117C diode array detector (DAD). Agilent Technologies 5977A GC/MSD was used for monosaccharide configuration determination. ^1^H and ^13^C nuclear magnetic resonance (NMR) data were acquired with a Bruker AVANCE 500 MHz spectrometer with a 5 mm triple resonance cryoprobe at 298 K. The referenced NMR solvent signals were used as acetone-*d*_6_, *δ*_H_ 2.05/*δ*_C_ 29.8, 206.2, and methanol-*d*_4_, *δ*_H_ 3.31, 4.87/*δ*_C_ 49.0, respectively. Heteronuclear multiple quantum correlation (HMQC) and heteronuclear multiple bond correlation (HMBC) experiments were optimized for 145 and 8 Hz, respectively. High-resolution electrospray ionization mass spectrometry (HRESIMS) data were recorded using an Agilent Accurate-Mass-Q-TOF LC/MS 6520 instrument equipped with an electrospray ionization (ESI) source. Fragmentor and capillary voltages were maintained at 125 and 3,500 V, respectively. Nitrogen was used as the nebulizing and drying gas (300°C) with a flow rate of 10 L/min, and the nebulizer pressure was set at 10 psi. Full-scan spectra were acquired across a scan range of *m/z* 100–1,000 at a rate of 1.03 spectra per second. Optical rotations were measured on an Anton-Paar MCP 200 polarometer. Circular dichroism (CD) spectra were recorded using a Chirascan spectropolarimeter.

#### 2.1.2 Chemicals, strain, and fermentation

The deuterium reagents were used from Cambridge Isotope Laboratories, Inc. (USA). The agar powder and PDA medium were used from Becton, Dickinson Co., Ltd. (USA). Yeast extracts and peptones were used from Oxoid, Inc. (UK). Driselase and PEG4000 were used from Sigma-Aldrich Trading Co., Ltd. (China). Miracloth was used from Merck-Calbiochem (Germany). The silica gel for column chromatography was used from Qingdao Haiyang Chemical Co., Ltd. (China). The monosaccharides and silanization reagents (*L*-cysteine methyl ester hydrochloride and *N*-trimethylsilylimidazole) were used from Shanghai Macklin Biochemical Technology Co., Ltd. (China). All molecular kits were purchased from Vazyme Biotech Co., Ltd. (China). All organic reagents were purchased from Thermo Fisher Scientific, Inc. (USA). The fungal strain *P. chlamydosporia* PC-170 was used for the study, and its solid (rice medium) fermentation method was the same as we described before (Qin et al., 2019).

### 2.2 Methods

#### 2.2.1 Extraction, isolation, and purification

The *P. chlamydosporia* PC-170 fermentation sample was extracted twice with ethyl acetate (EtOAc) and then recovered using a rotary evaporator to obtain the crude extract of 8.4 g. The crude extract was separated by reduced-pressure silica gel column chromatography using the gradient petroleum ether/EtOAc as the elution solvent, leading to 20 fractions named Fr1-Fr20 (EtOAc/petroleum ether at the following ratios: 0, 1, 3, 5, 8, 10, 15, 20, 25, 30, 35, 40, 45, 50, 55, 60, 70, 80, 90, and 100%, respectively). The 80 and 90% EtOAc eluted fractions (Fr18 and Fr19) were combined (100 mg) and further purified by RP-HPLC (Kromasil C_18_ column, 10 *μ*m, 10 × 250 mm, 30% MeCN in H_2_O over 15 min, 45% MeCN in H_2_O over 20 min, and then 100% MeCN over10 min, 2 mL/min) to afford 1 (4.3 mg, *t*_R_ 16.6 min), 3 (4 mg, *t*_R_ 30.1 min) and 2 (5.7 mg, *t*_R_ 37.9 min), respectively. The 20% EtOAc eluted fraction (200 mg) was purified by RP-HPLC (60% MeCN in H_2_O over 20 min, and then 100% MeCN over10 min, 2 mL/min) to afford 6 (5 mg, *t*_R_ 15.5 min) and 5 (4 mg, *t*_R_ 22.8 min), respectively. The 30% EtOAc eluted fraction (300 mg) was purified by RP-HPLC (45% MeCN in H_2_O over 20 min, and then 100% MeCN over 10 min, 2 mL/min) to afford 4 (5 mg, *t*_R_ 14.6 min).

Monocillin VI glycoside (**1**): white powder; [α]^25^_D_ + 122.98 (*c* 0.1, MeOH); UV (MeOH) *λ*_max_ (log *ε*), 225 (3.47), 260 (3.53), 313 (3.34) nm; CD (*c* 2.0 *μ*M, MeOH) *λ*_max_ (Δ*ε*), 207 (+19.18), 221 (−3.48), 230 (−0.23), 300 (+22.60) nm; ^1^H, ^13^C, and HMBC NMR data for **1** are listed in Table 1; HRESIMS, *m/z* 489.1737 [M + Na]^+^ (calculated for C_23_H_30_O_10_Na^+^, 489.1731).

**Table 1:** NMR data of compound 1 in methanol-*d*_4_.

Position	Monocillin VI glycoside (1)		
	*δ*_C_*^a^*, mult.	*δ*_H_*^b^* (*J* in Hz)	HMBC*^c^*
1	171.8, qC		
2	109.9, qC		
3	165.0, qC		
4	104.2, CH	6.64, d (2.5)	1, 2, 3, 5, 6
5	162.5, qC		
6	113.7, CH	6.44, d (2.5)	1, 2, 4, 5, 8
7	139.4, qC		
8	50.5, CH_2_	4.10, m 4.05, m	2, 6, 7, 9 2, 6, 7, 9
9	210.5, qC		
10	40.7, CH_2_	2.61, m 2.47, m	9, 11, 12 9, 11, 12
11	23.3, CH_2_	1.62, m	9, 10, 12, 13
12	25.9, CH_2_	1.65, m	9, 10, 11, 13, 14
13	31.9, CH_2_	2.20, m	11, 12, 14, 15
14	135.1, CH	5.72, dt (15.7, 7.2)	12, 13, 15, 16
15	130.9, CH	5.50, dd (15.7, 7.2)	13, 14, 16
16	75.5, CH	4.06, m	14, 15, 18
17	77.0, CH	5.26, m	1, 15, 16, 18
18	17.5, CH_3_	1.36, d (6.4)	16, 17
1’	101.6, CH	5.67, d (4.5)	5, 2′, 3′, 4’
2’	73.4, CH	4.20, dd (6.3, 4.5)	
3’	71.1, CH	4.10, m	1’, 2’, 4’, 5’
4’	88.0, CH	4.12, m	
5’	63.1, CH_2_	3.71, dd (12.4, 3.8) 3.64, dd (12.4, 3.8)	3’, 4′ 3′, 4’

^a^Recorded at 125 MHz. ^b^Recorded at 500 MHz. ^c^HMBC correlations are from proton (s) stated to the indicated carbons.

Colletogloeolactone A (**2**): white powder; [α]^25^_D_ + 123.98 (*c* 0.1, MeOH); UV (MeOH) *λ*_max_ (log *ε*), 216 (3.34), 262 (3.04), 302 (2.70) nm; CD (*c* 2.1 *μ*M, MeOH) *λ*_max_ (Δ*ε*), 207 (+7.83), 216 (−13.03), 225 (+7.88), 298 (+20.21) nm; HRESIMS, *m/z* 473.1798 [M + Na]^+^ (calculated for C_23_H_30_O_9_Na^+^, 473.1782).

Monocillin II glycoside (**3**): white powder; [α]^25^_D_ + 120.98 (*c* 0.1, MeOH); UV (MeOH) *λ*_max_ (log *ε*), 215 (3.43), 258 (3.14), 313 (2.71) nm; CD (*c* 2.0 *μ*M, MeOH) *λ*_max_ (Δ*ε*), 208 (+12.65), 237 (−27.87), 262 (+20.62), 316 (+8.03) nm; HRESIMS, *m/z* 471.1630 [M + Na]^+^ (calculated for C_23_H_28_O_9_Na^+^, 471.1626).

#### 2.2.2 Hydrolysis and determination of the absolute configuration of sugar moieties

Each compound (3 mg) was hydrolyzed by heating in MeOH (3 mL) and 3 M HCl (3 mL) at 70°C for 6 h. After removing MeOH and HCl by evaporation, the reaction mixture was diluted with H_2_O (10 mL) and extracted with EtOAc. The aqueous phase was evaporated in a vacuum to obtain the monosaccharide residue. The monosaccharide residue (1 mg) was heated in pyridine (1 mL) and 2 mg of *L*-cysteine methyl ester hydrochloride at 60°C for 2.5 h. The solvent was evaporated, and *N*-trimethylsilylimidazole (TSIM; 0.2 mL) was added to trimethylsilylate at 60°C for 2.5 h. The mixture was extracted using hexane and water (1 mL) (Lv et al., 2016). Similarly, the standard *D*-ribose and *L*-ribose were treated following the same procedure. The hexane extract obtained was subjected to gas chromatography–mass spectrometry (Agilent DB-1701 gas chromatographic column; 30 m × 250 *μ*m × 0.25 *μ*m; 1.5 mL/min) using the following conditions: injection temperature of 210°C; detector temperature of 250°C; initial column temperature of 200°C raised to 280°C at the rate of 60°C/min and maintained at 280°C for 35 min under N_2_ carrier gas.

#### 2.2.3 Nematicidal activity assay

The collection of J2 nematodes was carried out according to a previously described procedure (Yin et al., 2021). The concentration of nematodes was adjusted to 1,000 strips/mL using sterile water for observation under a microscope. The abamectin (50 *μ*g/mL) was used as the positive control, while the concentrations tested for fractions Fr1-Fr20 were 2 mg/mL. The lethal concentration 50% (LC_50_) of the compounds was determined. The compounds were prepared in five concentrations: 10 mg/mL, 7 mg/mL, 5 mg/mL, 2.5 mg/mL and 1.25 mg/mL. Each well contained 100 *μ*L (5 *μ*L sample added to 95 *μ*L nematodes) with three biological and five technical repetitions (Seo et al., 2014). The 96-well plates were incubated at 28°C, and nematode morphology was assessed under a microscope after 24 h. Nematodes were stimulated with 0.5 M NaOH and were considered dead if they remained in a rigid morphology. Regression analysis was conducted using logarithmic values of compound concentrations and their corresponding corrected mortality values. Based on the regression curve equations, the LC_50_ of the compounds was calculated. Mortality and corrected mortality rates were computed as follows:

$$\mathrm{The}\;\mathrm{mortality}\;\mathrm{rate}=\frac{\mathrm{number}\;\mathrm{of}\;\mathrm{nematode}\;\mathrm{deaths}}{\mathrm{total}\;\mathrm{number}\;\mathrm{of}\;\mathrm{nematodes}}\times 100\%$$

$$\mathrm{The}\;\mathrm{corrected}\;\mathrm{mortality}\;\mathrm{rate}=\frac{\left(\begin{array}{l} \mathrm{treatment}\;\mathrm{mortality}\;\mathrm{rate}\\ -\mathrm{control}\;\mathrm{mortality}\;\mathrm{rate} \end{array}\right)}{\left(1-\mathrm{control}\;\mathrm{mortality}\;\mathrm{rate}\right)}\times 100\%$$

#### 2.2.4 DNA isolation, vector construction, and gene knockout

The genomic DNA of wild-type *P. chlamydosporia* PC-170 was extracted using FastPure Microbiome DNA Isolation Kit (Vazyme, China). The upstream arm (1,500 bp) and downstream arm (1,500 bp) of the knockout gene were amplified using the high-fidelity 2 × Phanta Flash Master Mix (Vazyme, China) with PC-170 genomic DNA as a template to obtain the UP and DOWN fragments. The pUC-Cas9-neo vector (Luo et al., 2023) was used as the template for amplify the NEO-resistant fragment. All amplicons were combined and ligated into pUC19 (Supplementary Figure S7A) using ClonExpress MultiS One Step Cloning Kit (Vazyme, China) to construct the knockout vector (Supplementary Figure S7B).

The knockout of targeted genes was performed through the PEG-mediated transformation of fungal protoplasts (Supplementary Figure S7C; Bok and Keller, 2004; Li et al., 2006). Freshly germinated conidia were collected on Miracloth, rinsed with 0.7 M NaCl, and digested with 20 mg/mL driselase for 3.5 h. Enzyme lysis products were filtered by Miracloth and rinsed with STC buffer (145.6 g/L Sorbitol, 7.4 g/L CaCl_2_·2H_2_O, 1.6 g/L Tris–HCl). The protoplast concentration was adjusted to 1 × 10^7^ with STC buffer. About 120 *μ*L of protoplast was incubated with 5 *μ*g of linearized DNA which was supplemented with STC buffer to 60 *μ*L on ice for 20 min. Then, 160 *μ*L of PEG4000 was added and incubated at room temperature for 15 min. Total 20 *μ*L of the protoplasts treated as above was transferred into 5 mL of T-TOP medium (0.5 g/L KCl, 0.5 g/L MgSO_4_·7H_2_O, 1.0 g/L KH_2_PO_4_, 2.0 g/L NaNO_3_, 200.0 g/L sucrose, 20.0 g/L glucose, 10 g/L agar powder) and then spread evenly on PDA dishes. After cultured at 28°C for 24 h, the plates were covered with 10 mL T-TOP containing 400 *μ*g/mL G418. After incubation at 28°C for 3–5 days, the transformants were transferred to new PDA plates with G418. All transformants were sub-cultured for 3 generations to obtain stable transformants. All mutants were identified by diagnostic PCR. All primers and plasmids used in this study are described in Supplementary Table S1.

## 3 Results

### 3.1 *In vitro* nematicidal activity of the crude extract from *Pochonia chlamydosporia* PC-170

**Figure 1:**
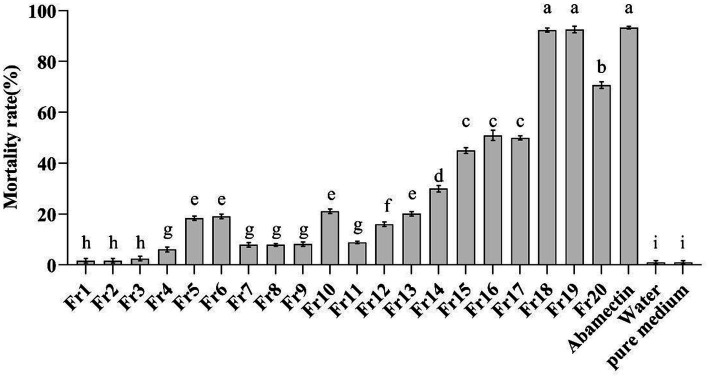
*In-vitro* test of the mortality of the J2s of *M. incognita*. Fr1-Fr20 (2 mg/ml) were as the treatment group, abamectin (50 μg/ml) was as the positive control, and water and the pure medium extract were as the negative control. The error bars represent SEs (*n* = 15) for three biological and five technical repetitions and different lowercase letters indicate significant differences between treatments (*p* < 0.05).

The *in vitro* nematicidal activity of the crude extract from *P. chlamydosporia* PC-170 was evaluated on *M. incognita* J2s mortality. The EtOAc crude extract (8.4 g) was separated by reduced-pressure silica gel column chromatography to get 20 fractions (Fr1-Fr20). After *in vitro* nematicidal activity test, fractions Fr18 and Fr19 showed the highest nematicidal activity (mortality rates at 92.4 and 92.6%, respectively), in line with that of the positive control abamectin (50 *μ*g/mL) against J2s of *M. incognita* (mortality rates at 93.3%), while other fractions showed a relative lower nematicidal activity, and the crude extract from the pure medium showed no nematicidal activity (Figure 1). This result indicated that some natural products in the crude extract of *P. chlamydosporia* PC-170 had a nematicidal activity that required further isolation and identification.

### 3.2 Purification and structural identification of RALs

**Figure 2:**
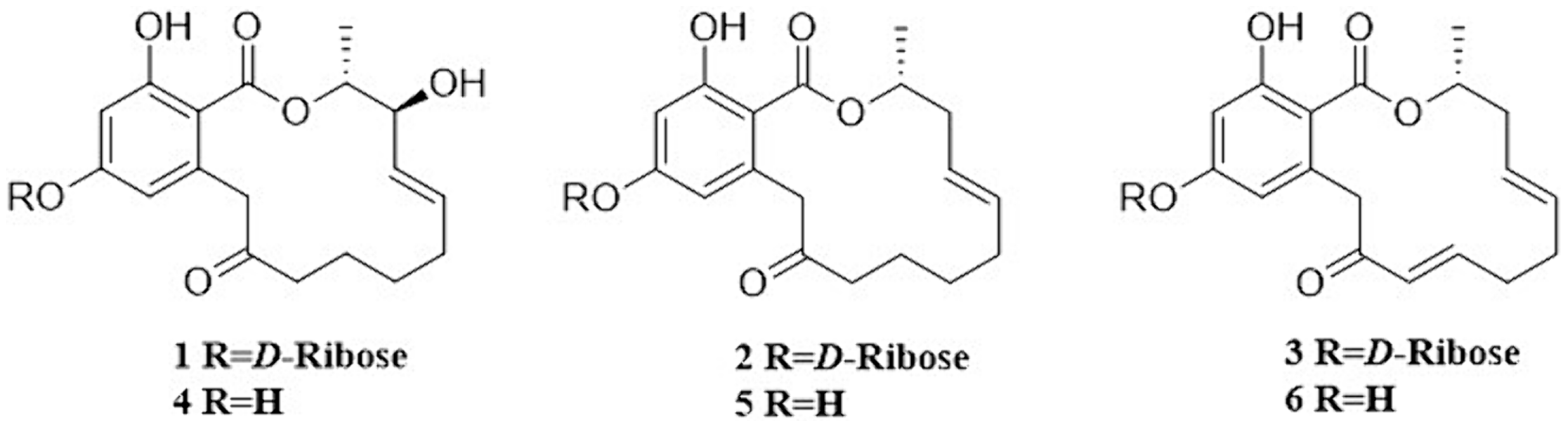
Structures of compounds **1–6**.

Six RALs compounds were isolated by RP-HPLC from the nematicidal activity fractions. The known compounds **2**–**6** (Figure 2) were identified as colletogloeolactone A (**2**) (Kim et al., 2023), monocillin II glycoside (**3**) (Hellwig et al., 2003), monocillin VI (**4**) (Qin et al., 2019), monocillin IV (**5**) (Ayer et al., 1980) and monocillin II (**6**) (Ayer et al., 1980) respectively, by comparison of their NMR (Supplementary Figures S2–S6) and MS data (Supplementary Figures S8B–S8F) to those previously reported.

**Figure 3:**
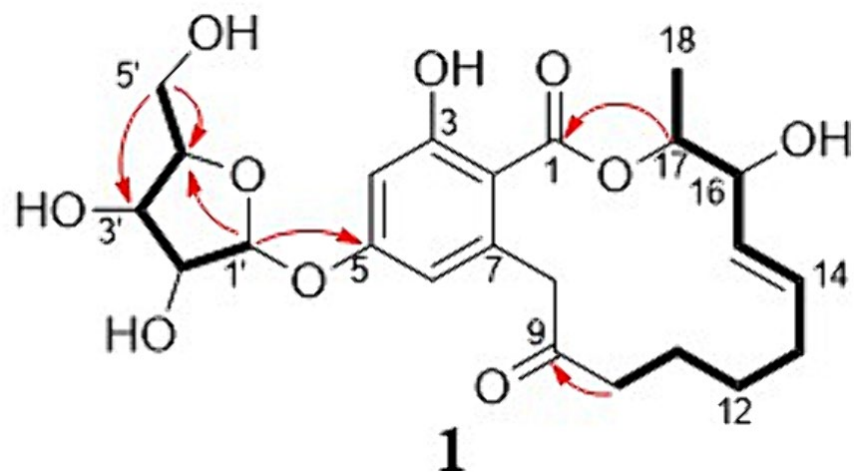
Selected key HMBC (H → C) and ^1^H–^1^H COSY (─) correlations of compound 1.

**Figure 4:**
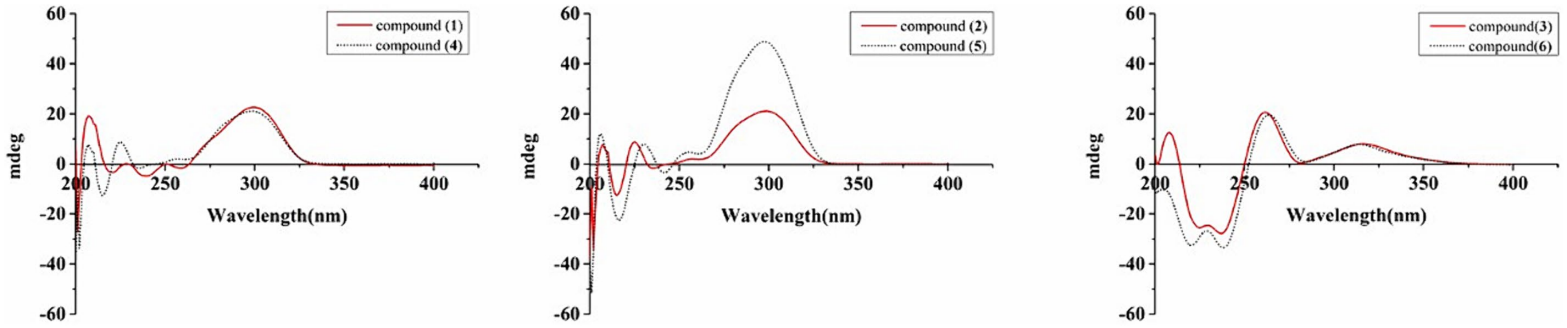
CD spectra of compounds **1–6**.

**Figure 5:**
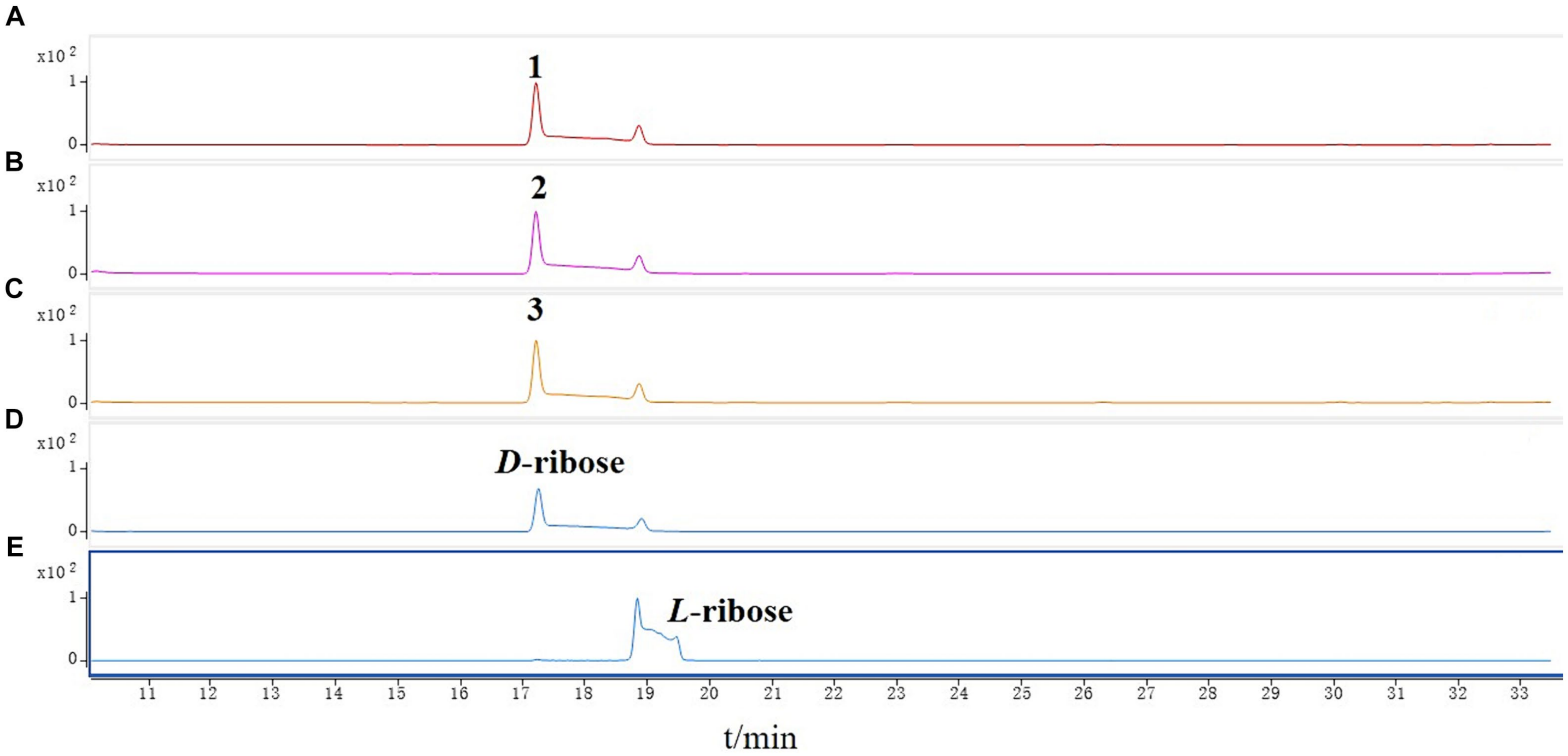
GC–MS analysis of the silylated monosaccharide of **(A)** monocillin VI glycoside (**1**), **(B)** colletogloeolactone A **(2)**, **(C)** monocillin II glycoside **(3)**, **(D)** *D*-ribose, **(E)** *L*-ribose.

The new compound monocillin VI glycoside (**1**) was assigned the molecular formula C_23_H_30_O_10_ (nine degrees of unsaturation) based on HRESIMS (Supplementary Figure S8A, *m/z* 489.1737 [M + Na]^+^). Analysis of its ^1^H, ^13^C, and HMBC NMR spectroscopic data (Table 1; Supplementary Figure S1) revealed the presence of one methyl group, five methylenes, two oxymethines, eight aromatic/olefinic carbons (four of which were protonated and two of which were attached to oxygen), one carboxyl carbon (*δ*_C_ 171.8), one ketone carbon (*δ*_C_ 210.5), and one pentose sugar unit. Its NMR spectrum feature revealed that **1** possessed similar fragments to those of other RAL analogs (Figure 2). Interpretation of the ^1^H–^1^H correlation spectroscopy (COSY) and HMBC NMR data (Figure 3) for **1** established its main structure as a 14-membered resorcylic acid lactone ring, which was an analog of the known compound monocillin VI (**4**) (Qin et al., 2019), a metabolite that was co-isolated from this extract. Comparison of the NMR spectroscopic data of **1** (Table 1) with those of **4** suggested that they possess the same monocillin VI moiety, but **1** contains one more pentose sugar substituted unit. ^1^H–^1^H COSY and HSQC data of **1** identified an isolated spin-system of C-1’–C-5′, and HMBC correlations from H_2_-5′ to C-3′ and C-4′, and from H-1′ to C-4′, confirmed the pentose sugar moiety (C-1’–C-5′), whereas a key HMBC correlation from H-1′ to C-5 indicated that the pentose sugar moiety must be attached to C-5 of the 2,4-dihydroxybenzoate unit. On the basis of these data, the gross structure of monocillin VI glycoside was established, as shown in **1**. The geometry of the disubstituted double bond (C-14/C-15) in the side chain was deduced to be *trans* from the large coupling constant (*J*_14,15_ = 15.7 Hz) of the olefinic protons. The absolute configurations of the C-16 secondary alcohol and C-17 in **1** were assigned to be the same as that of compound **4** because of the identical CD curves between compounds **1** and **4** (Figure 4) and the positive specific rotation values for both compounds **1** and **4**. Sugar hydrolysis experiment (Lv et al., 2016) was applied to assign the absolute configuration of the pentose sugar residue resulting from acid hydrolysis of monocillin VI glycoside (**1**). The pentose sugar residue present in the acid hydrolyzate of **1** was analyzed by GC–MS along with those of authentic *D*- and *L*-pentose sugars (Figure 5). After analysis, the pentose sugar residue in **1** was determined to be *D*-ribose.

Structurally, the known compounds colletogloeolactone A (**2**) and monocillin II glycoside (**3**) were the glycosylation derivatives of compounds monocillin IV (**5**) and monocillin II (**6**), respectively. However, the absolute configurations of the pentose sugar residues in **2** and **3** were still unknown. We applied the sugar hydrolysis experiment and GC–MS analysis method to determine both of the pentose sugar residues in **2** and **3** were also *D*-ribose (Figure 5). Therefore, the structures of **2** and **3** were determined to be 5-*D*-ribose monocillin IV and 5-*D*-ribose monocillin II, respectively.

### 3.3 Nematicidal activity of RALs

All of these compounds **1–6** were tested for nematicidal activity against *M. incognita.* The glycosylated compounds **1–3** showed a modest inhibitory effect, with LC_50_ values of 94, 152, and 64 *μ*g/mL, respectively. The non-glycosylated compounds **4–6** showed no nematicidal activity above 1,000 *μ*g/mL (Supplementary Table S2).

### 3.4 Identification of the key biosynthetic genes and validation of mutant strains

**Figure 6:**
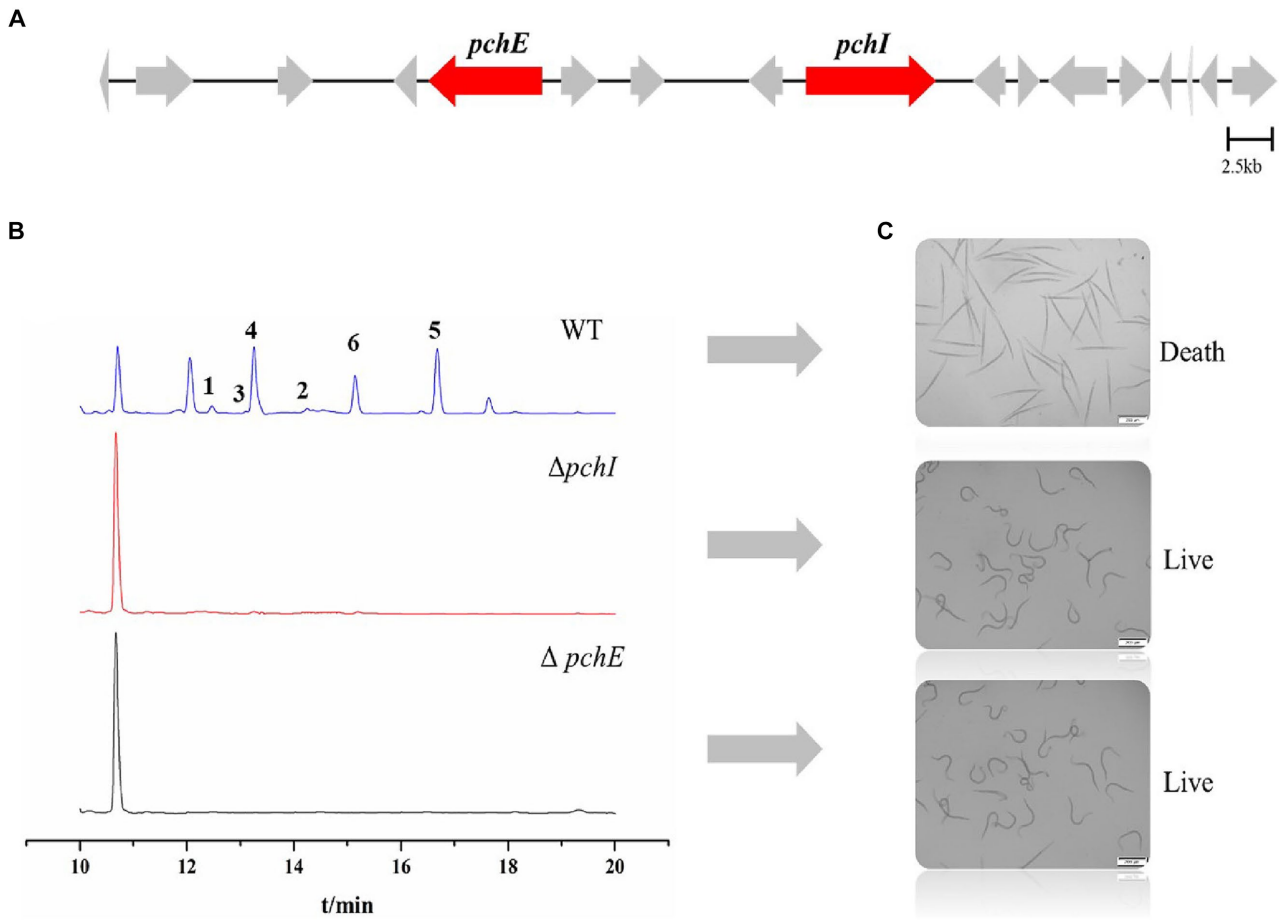
Identification of core genes of *P. chlamydosporia* strain PC-170 and nematicidal activity of Δ*pchI* and Δ*pchE*. **(A)** The biosynthetic gene cluster for RALs compounds in *P. chlamydosporia* strain PC-170, two key genes. **(B)** HPLC analysis of rice fermentation crude extract from the strains wild-type (WT), Δ*pchI*, and Δ*pchE* of *P. chlamydosporia* PC-170. **(C)** Nematicidal activity of rice fermentation crude extract from the strains wild-type (WT), Δ*pchI*, and Δ*pchE* of *P. chlamydosporia* PC-170.

In a previously published paper, we predicted the gene cluster for the synthesis of RALs compounds in *P. chlamydosporia* PC-170 (Qin et al., 2019). Among them, the *pchE* (nrPKS) and *pchI* (hrPKS) genes were proposed to be the two key biosynthetic genes (Figure 6A). Herein, we successfully constructed the knockout vectors pUC19-PcSM08964 and pUC19-PcSM08959 and obtained mutant strains Δ*pchE* and Δ*pchI* through homologous recombination (Supplementary Figure S9). The crude extracts of the wild-type and mutant strains were analyzed by HPLC under the same conditions. The results showed that all six compounds, including the nematicidal glycosylated compounds, disappeared in the mutant strains compared to the wild-type (Figure 6B). Additionally, the nematicidal activity of the mutant strains also disappeared (Figure 6C). Based on the above results, the RALs biosynthesis gene cluster was validated, and the two PKS genes, *pchE* and *pchI*, were confirmed to be the RALs biosynthetic core genes (Figure 6).

## 4 Discussion

The nematicidal activity of six RALs compounds was tested using the same method. It was found that only the glycosylated compounds were nematicidal, while those non-glycosylated compounds were not. We speculate that glycosylation plays a key role in nematicidal activity. The gene cluster for the synthesis of RALs compounds in *P. chlamydosporia* PC-170 has been identified. A total of 30 RALs compounds have been discovered in *P. chlamydosporia* (Khambay et al., 2000; Hellwig et al., 2003; Shinonaga et al., 2009a,b; Qin et al., 2019). RALs compounds have a range of biological activities, but less nematicidal activity has been reported. Studies have shown that 10, 11-Dehydrocurvularin exhibits nematicidal activity (Zhang et al., 2017), which may be related to its heat shock response and immune-modulatory activities (Xu et al., 2013). Glycosylation of natural products is an important resource for developing lead compounds in the field of pesticides (Kohara et al., 2007). Glycosylation has the potential to enhance the polarization and water-solubility of compounds, thereby influencing their intracellular and intercellular transportation (Moradi et al., 2016). For example, the anticancer properties of paclitaxel face a significant obstacle in development due to its limited solubility. The monoglycosylated and double-glycosylated derivatives exhibit 49.8-fold and 97.4-fold, respectively, greater aqueous solubility than paclitaxel, and preserve their higher efficacy (Mao et al., 2018). Glycosylation not only changes the physicochemical properties of a compound but also influences its biological activity (Chen, 2011; Hofer, 2016). For another example, megalomycin (glycosylated erythrocin D) exhibits notable antiviral and antiparasitic activities, but erythrocin D itself does not possess similar biological activities (Useglio et al., 2010). These findings are consistent with the results of the current research.

It is crucial to explore the genes related to glycosylation in *P. chlamydosporia* PC-170. While glycosyltransferase genes in plants and bacteria have been extensively researched, little is known about glycosyltransferase genes in fungi, most of which are glucosyltransferases (Tiwari et al., 2016; Xie et al., 2018). There are no genes encoding glycosyltransferase functions on the RAL synthesis gene cluster of *P. chlamydosporia* PC-170, but analysis of the *P. chlamydosporia* 170 genome data revealed 118 glycosyltransferases, and the glycosylation products are formed by catalysis of glycosyltransferases outside the gene cluster (Lin et al., 2018). For example, the glycosylation of phenolic compounds is catalyzed by the glycosyltransferase MhGT1, identified from *Mucor hiemalis* (Feng et al., 2017). Furthermore, a novel glycosyltransferase (BbGT86) was identified from *Beauveria bassiana* ARSEF 2860 using bioinformatics and genome mining, and the glycosylation of desmethyl-lasiodiplodin (DLD) was achieved by heterologous expression (Xie et al., 2018). There is still no discovery of *D*-ribosyltransferases in fungi. Therefore, the subsequent plan is to identify genes with glycosyl transfer function in *P. chlamydosporia* PC-170 and assess the impact of glycosylation on various RALs products, aiming to discover new natural products with potent nematicidal activity against *M. incognita*.

## 5 Conclusion

In conclusion, we isolated and characterized six resorcylic acid lactones (RALs) from the nematicidal biocontrol fungus *P. chlamydosporia* PC-170, identifying three glycosylated RALs with potent nematicidal activity and three non-glycosylated RALs with antibacterial properties. The novel compound, monocillin VI glycoside (**1**), was elucidated using high-resolution electrospray ionization mass spectrometry (HRESIMS) and nuclear magnetic resonance (NMR) data, with its monosaccharide configuration confirmed through sugar hydrolysis and gas chromatography–mass spectrometry (GC–MS) analysis. Further, the identification of two biosynthetic polyketide synthase (PKS) genes, *pchE* and *pchI*, was achieved via gene knockout experiments. Notably, the glycosylated RALs **1**–**3** demonstrated significant nematicidal activity against *M. incognita*, with LC_50_ values of 94, 152, and 64 *μg*/mL, respectively, underscoring their potential for developing new nematicidal agents against *M. incognita*.

## Data availability statement

The datasets presented in this study can be found in online repositories. The names of the repository/repositories and accession number(s) can be found at: https://www.ncbi.nlm.nih.gov/, ASM165323v2.

## Author contributions

ZL: Investigation, Writing – original draft. NL: Data curation, Investigation, Writing – original draft. WZ: Formal analysis, Investigation, Writing – review & editing. RAAK: Data curation, Writing – review & editing. JL: Conceptualization, Writing – original draft. JZ: Formal analysis, Methodology, Writing – original draft. YY: Writing – original draft. ZM: Conceptualization, Writing – review & editing. BX: Conceptualization, Writing – review & editing. LZ: Conceptualization, Writing – review & editing. YL: Conceptualization, Supervision, Writing – review & editing.
