# Supplementary material for: Nematicidal glycosylated resorcylic acid lactones from the fungus Pochonia chlamydosporia PC-170 and their key biosynthetic genes
Source: Front Microbiol. 2024 Apr 4;15:1385255. doi: 10.3389/fmicb.2024.1385255 (PMC11024724; doi:10.3389/fmicb.2024.1385255)
Supplement: Supplementary file 1 [file Data_Sheet_1.pdf]

Supplementary Material.

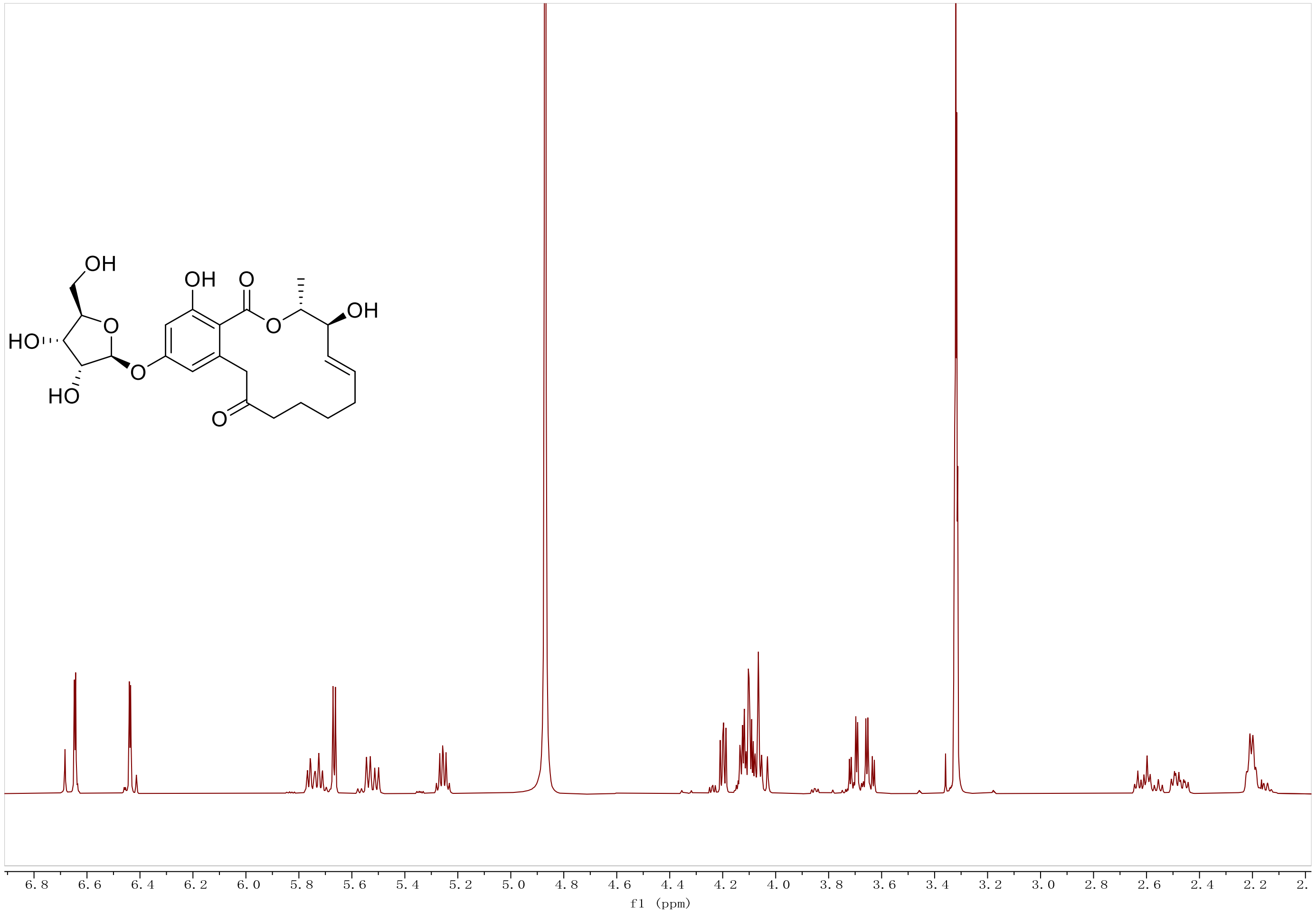

Figure S1. **NMR spectra of monocillin VI glycoside (1)**

a. ^{1}H NMR spectrum of monocillin VI glycoside (**1**; 500MHz, Methanol-d_4)

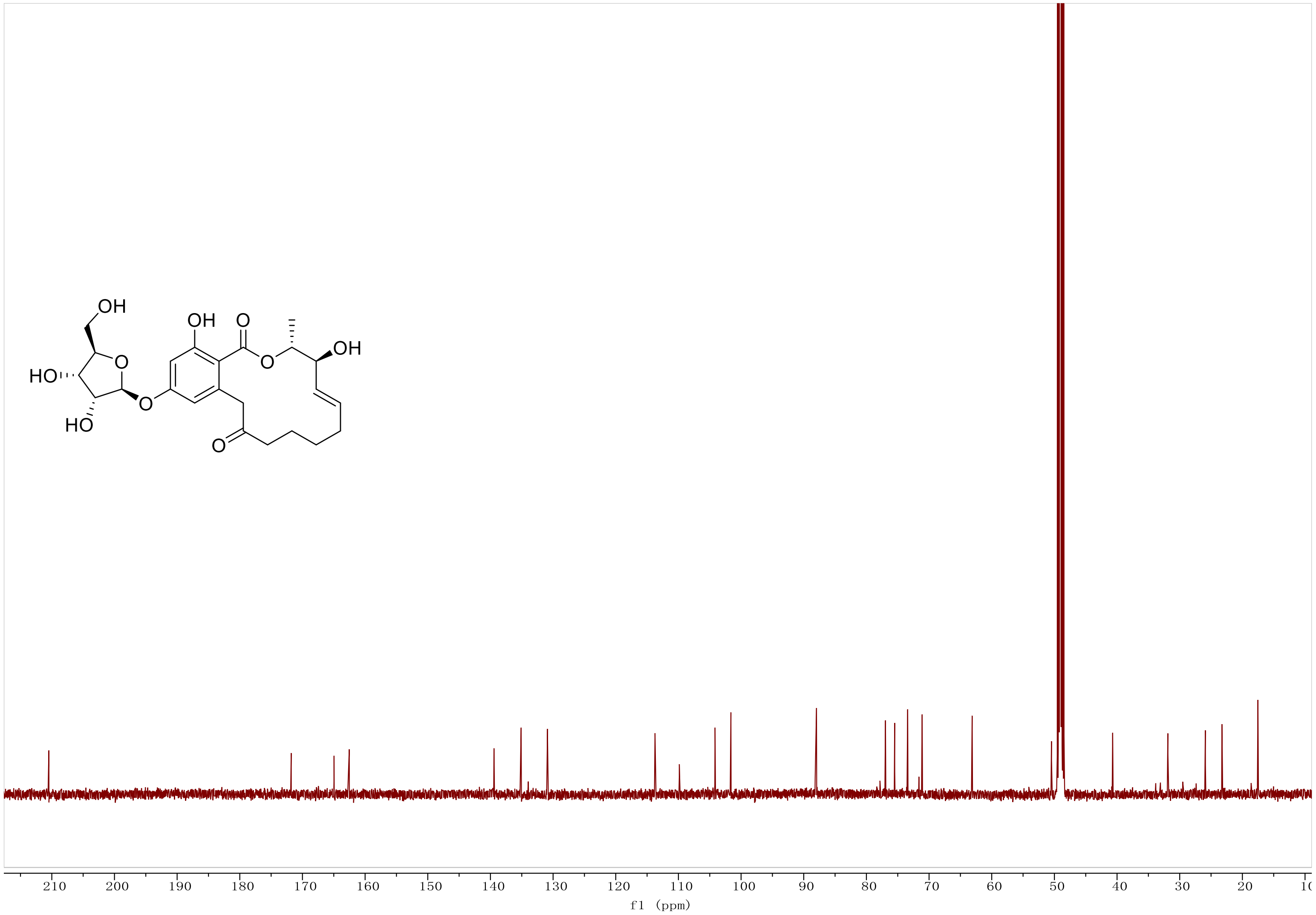

b. ^{13}C NMR spectrum of monocillin VI glycoside (**1**; 125MHz, Methanol-d_4)

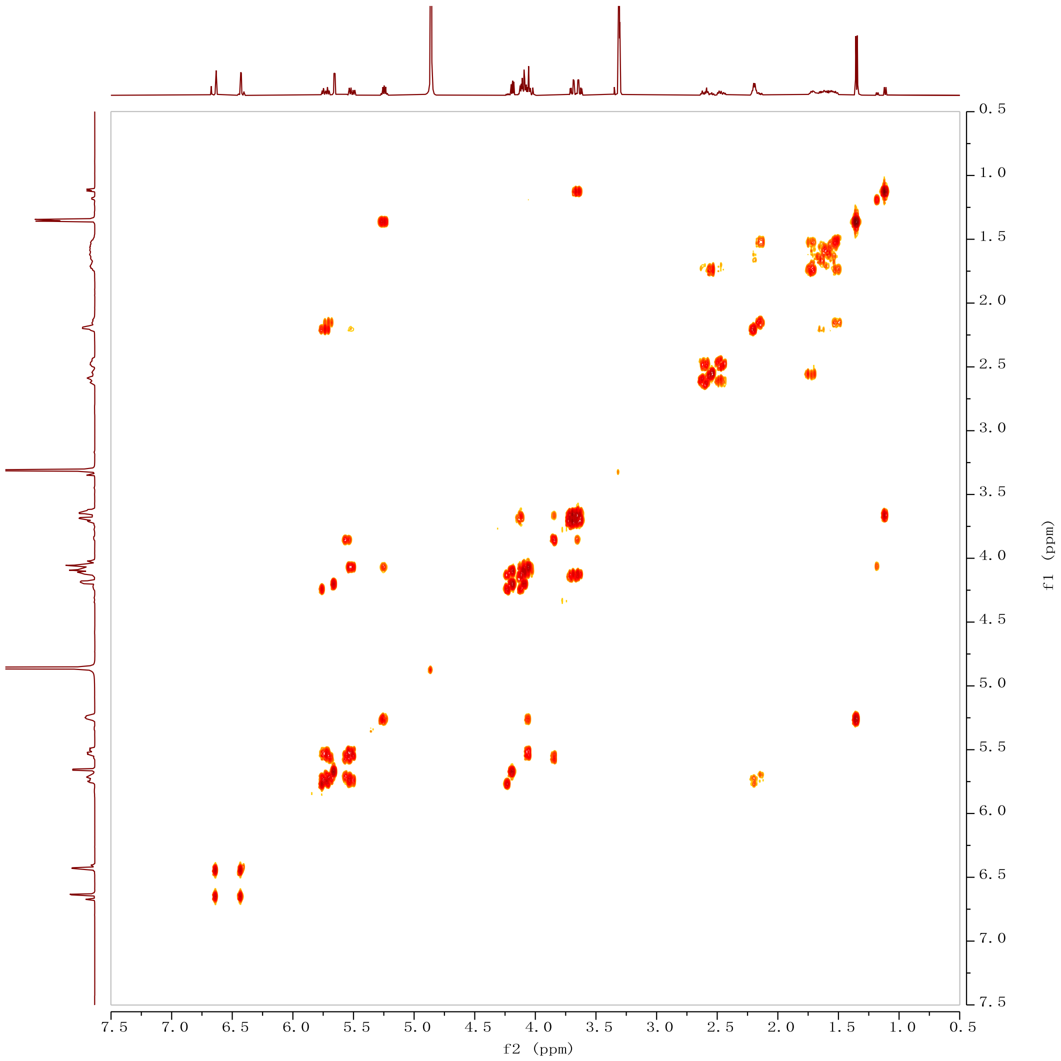

c. The 2D NMR ^1H–^1H COSY of monocillin VI glycoside (**1**)

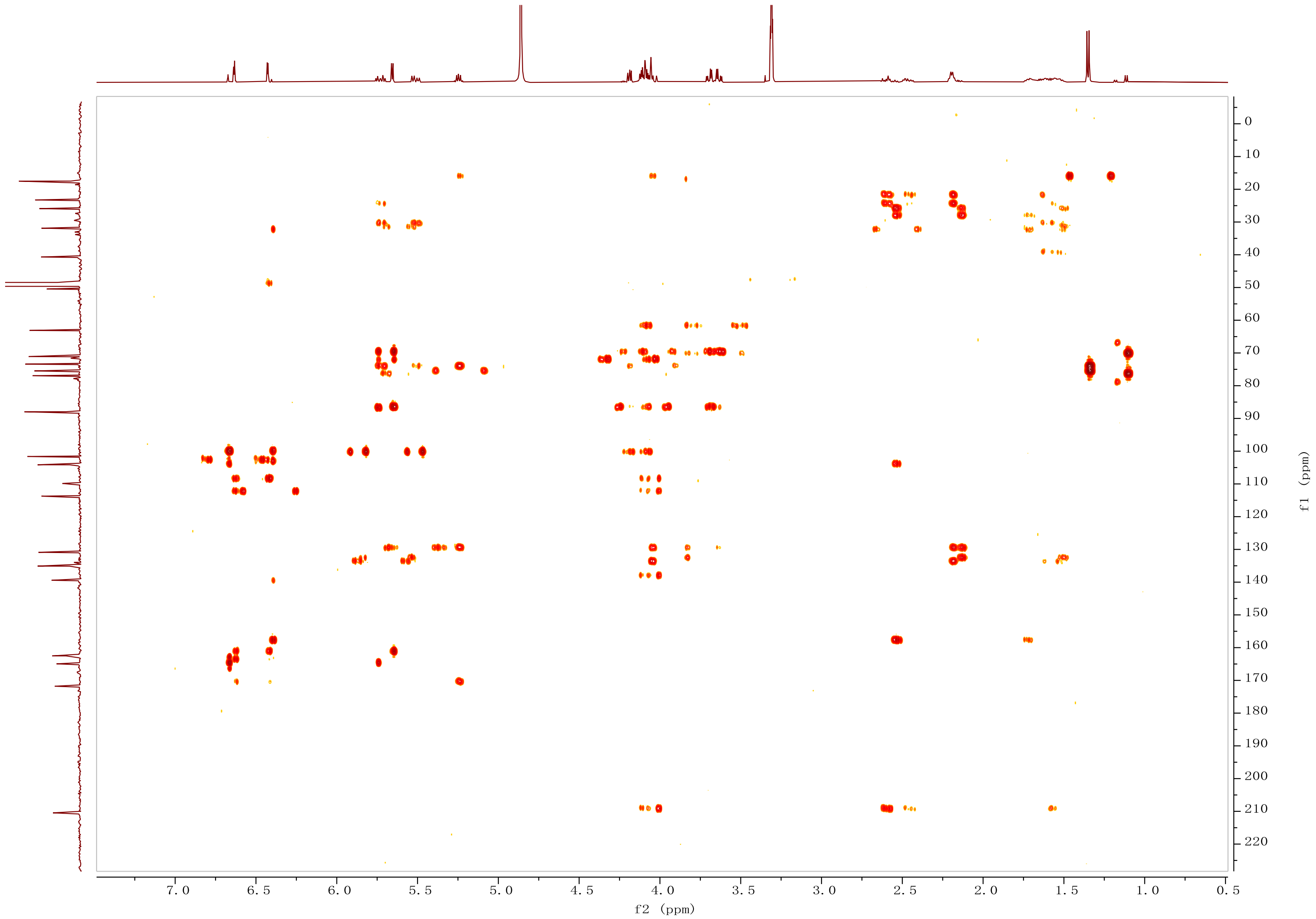

d. The 2D NMR HMBC of monocillin VI glycoside (**1**)

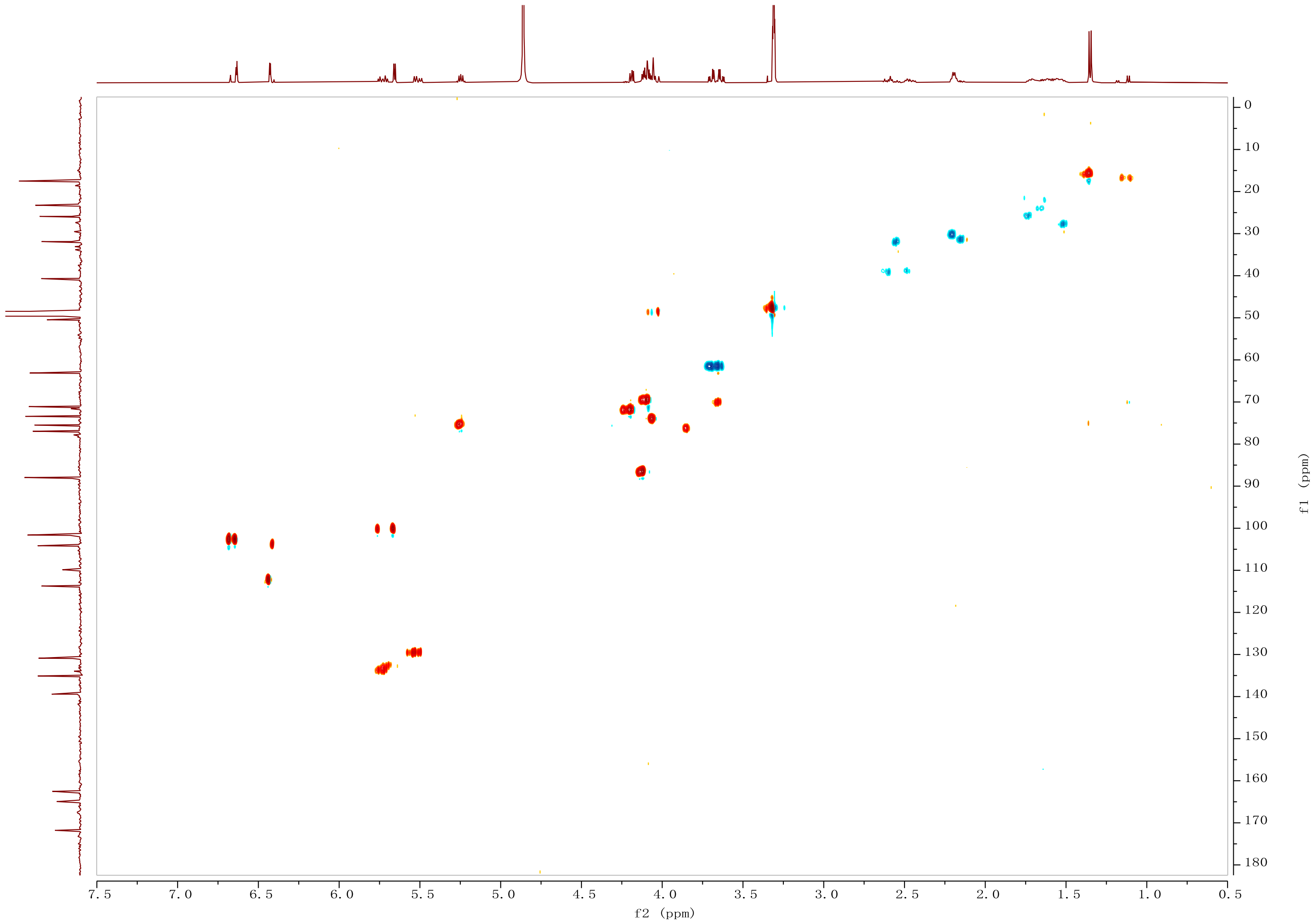

e. The 2D NMR HSQC of monocillin VI glycoside (**1**)

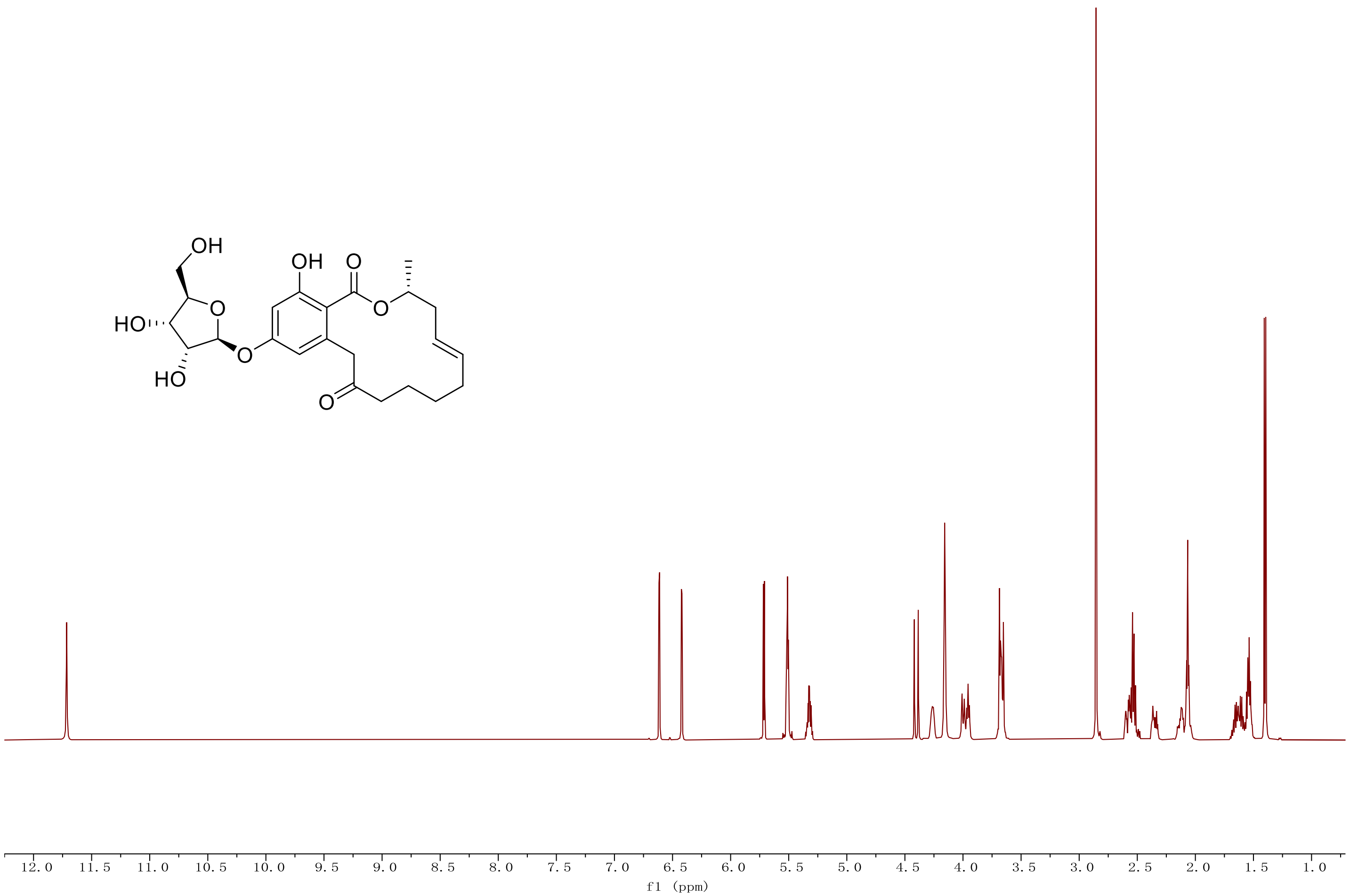

Figure S2. NMR spectra of colletogloeolactone A (2)

a. ¹H NMR spectrum of colletogloeolactone A (**2**; 500MHz, Acetone-d_6)

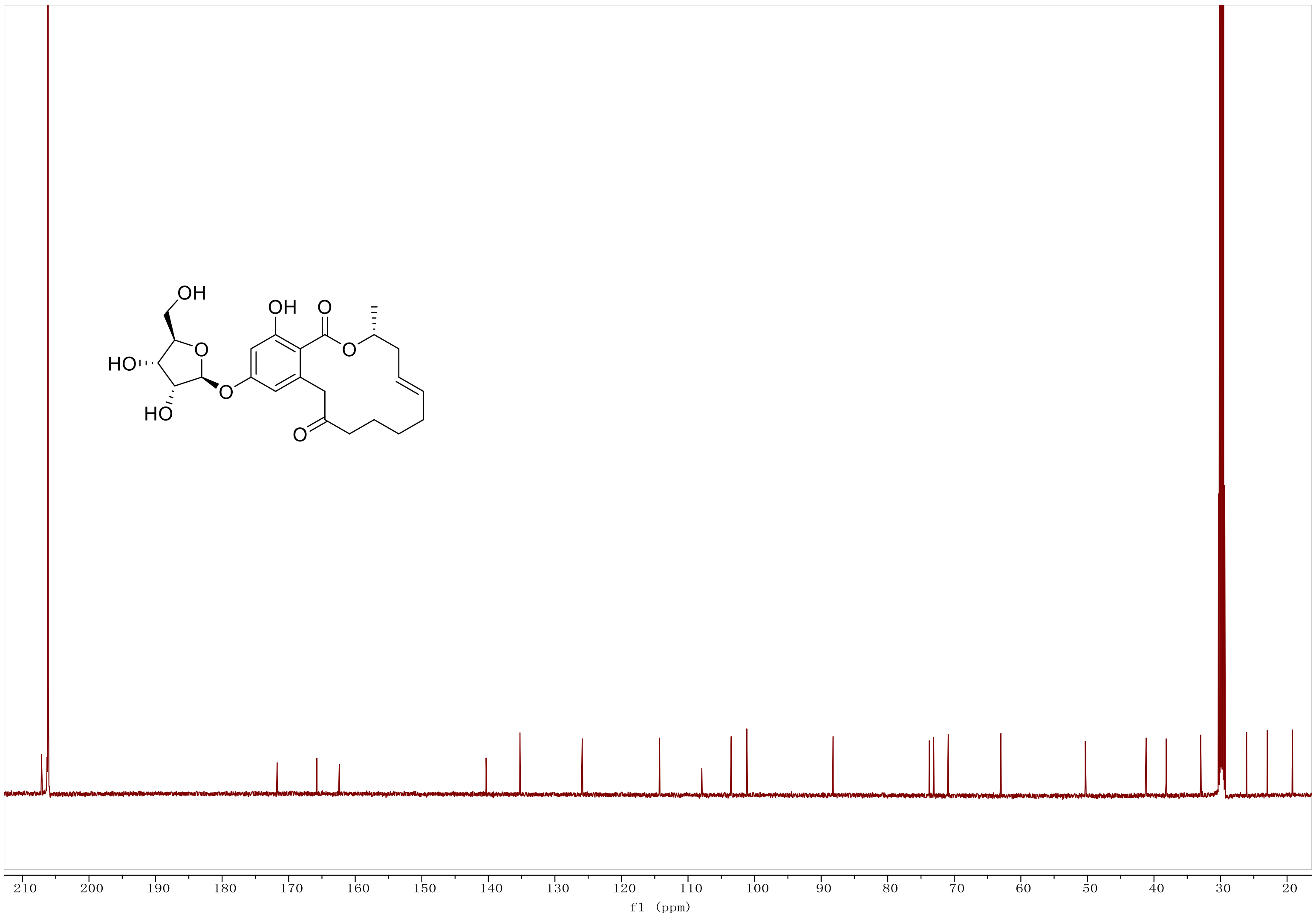

b. ^{13}C NMR spectrum of colletogloeolactone A (**2**; 125MHz, Acetone-d_6)

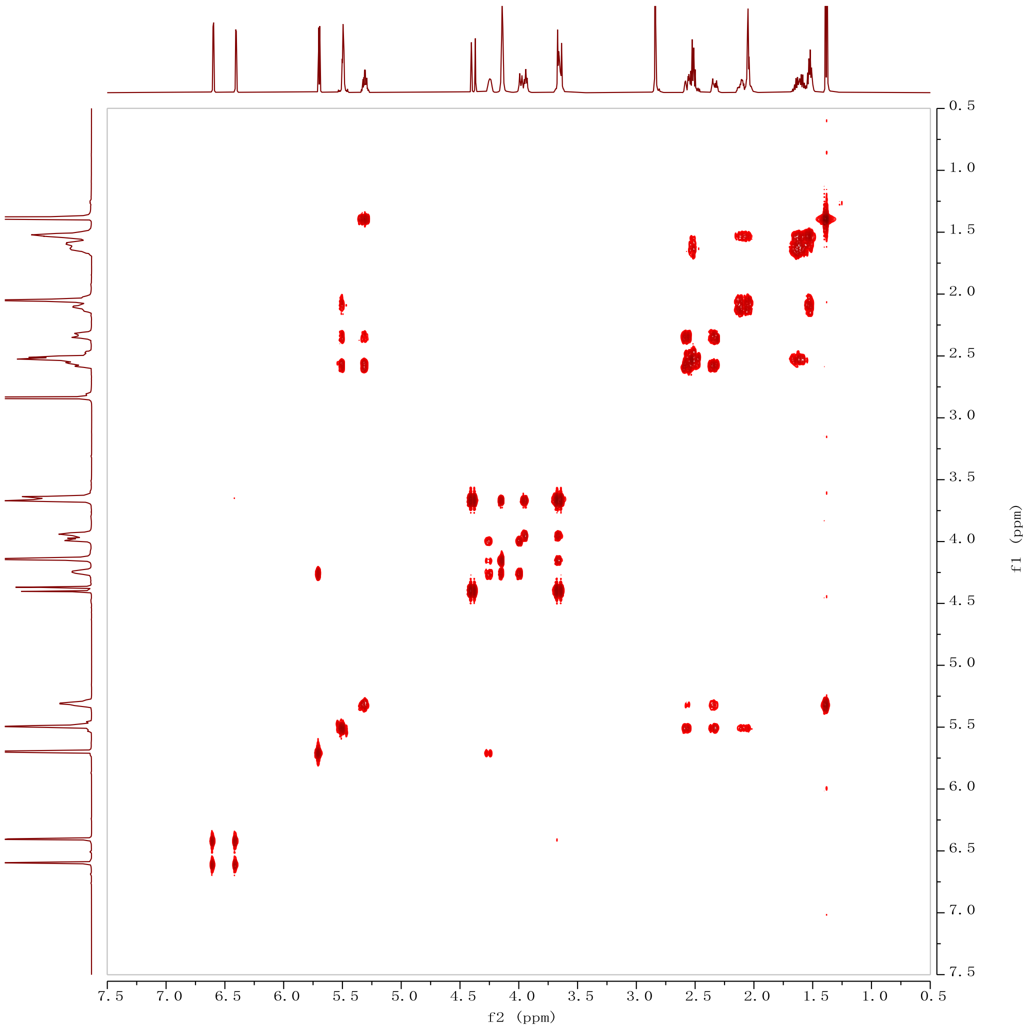

c. The 2D NMR ^{1}H–^{1}H COSY of colletogloeolactone A (**2**)

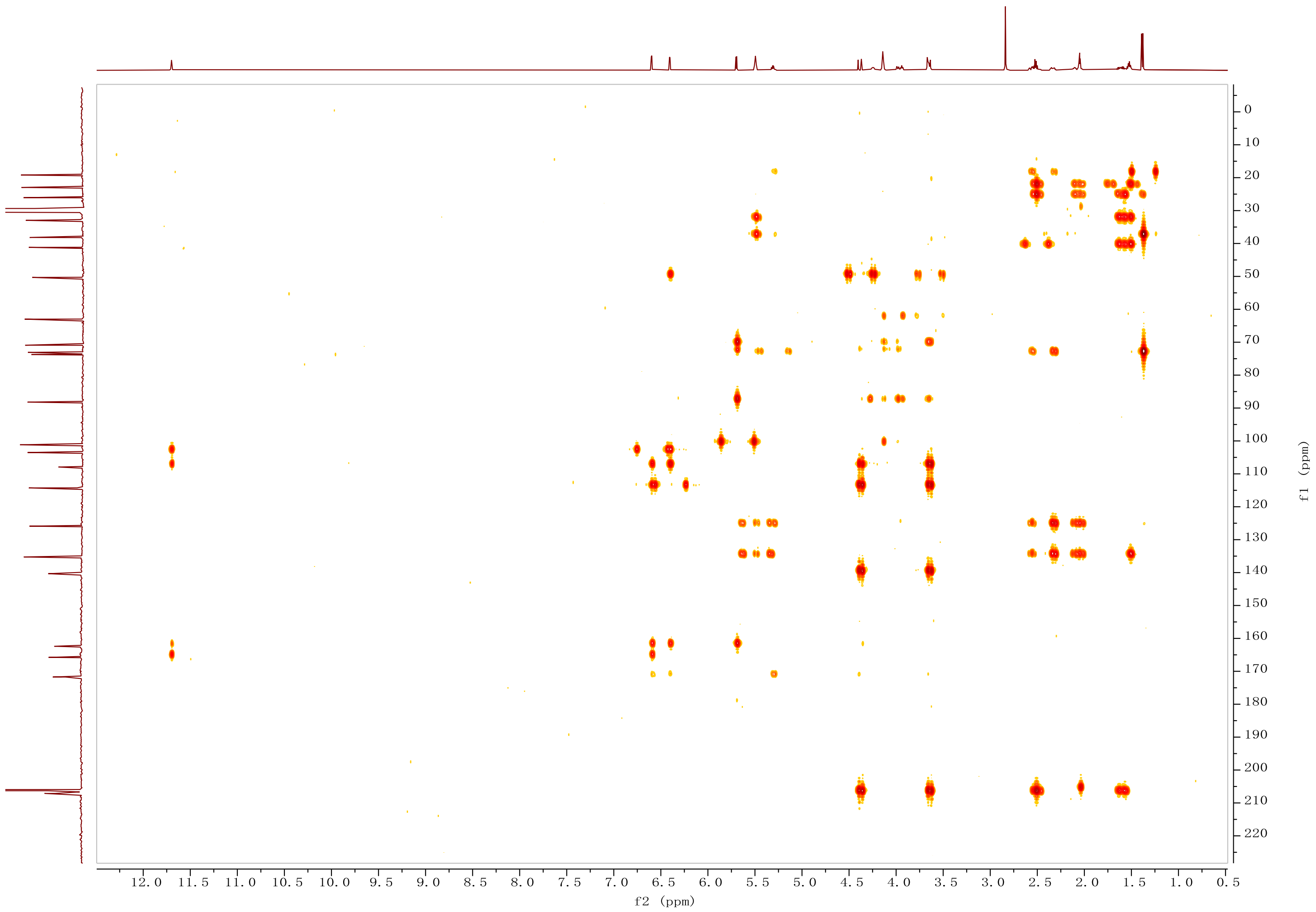

d. The 2D NMR HMBC of colletogloeolactone A (**2**)

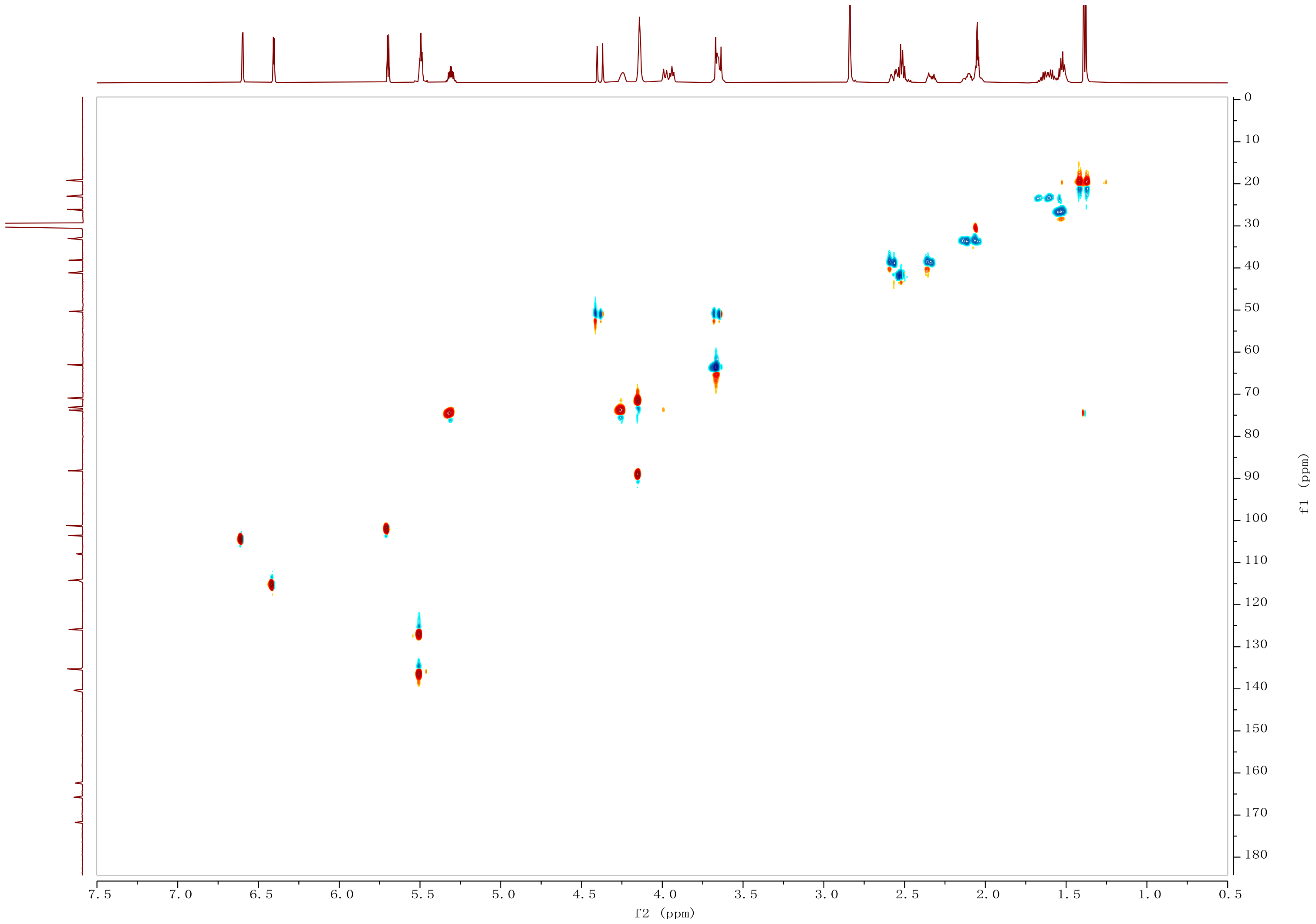

e. The 2D NMR HSQC of colletogloeolactone A (**2**)

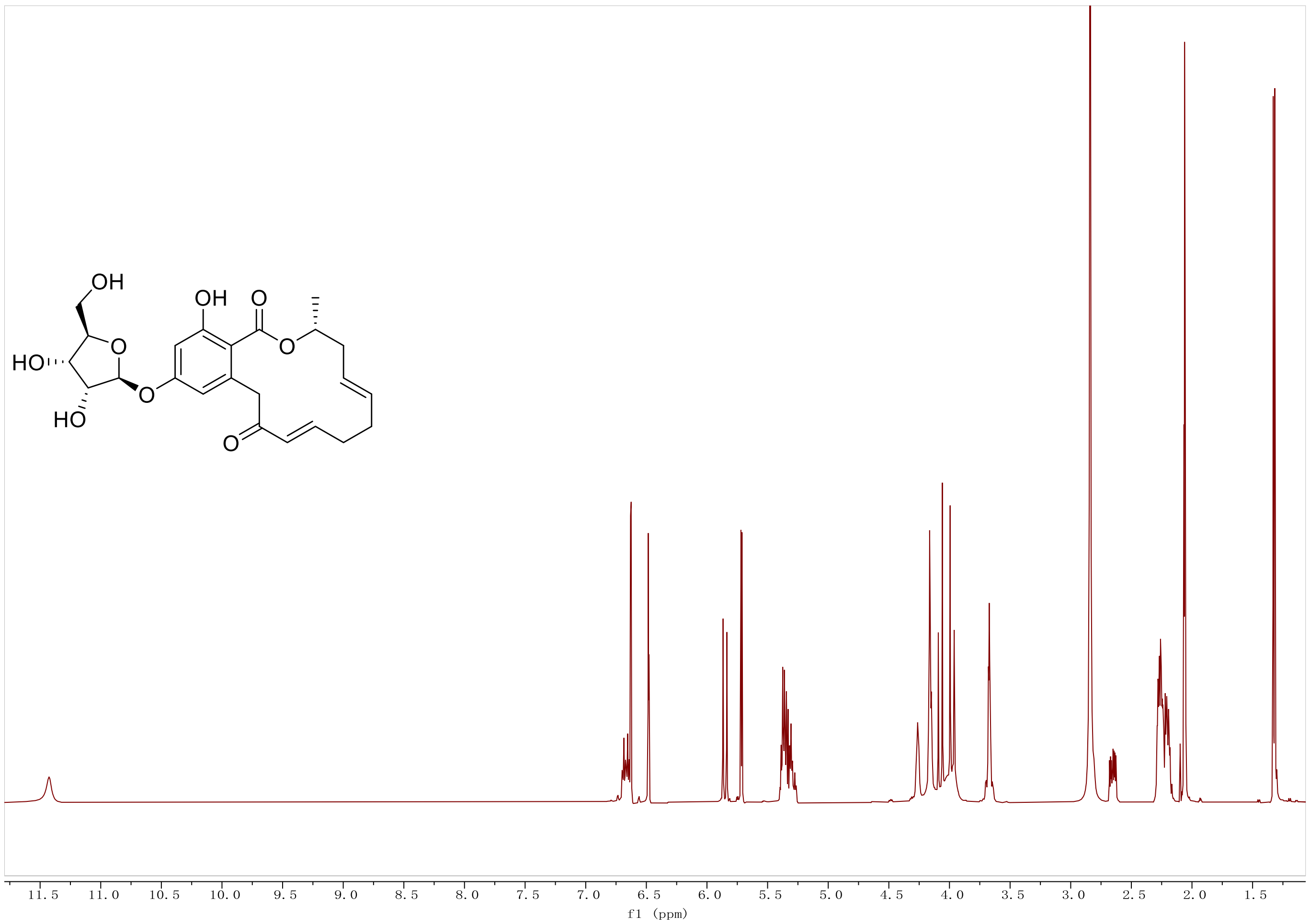

Figure S3. NMR spectra of monocillin II glycoside (3)

a. ^{1}H NMR spectrum of monocillin II glycoside (**3**; 500MHz, Acetone-d_6)

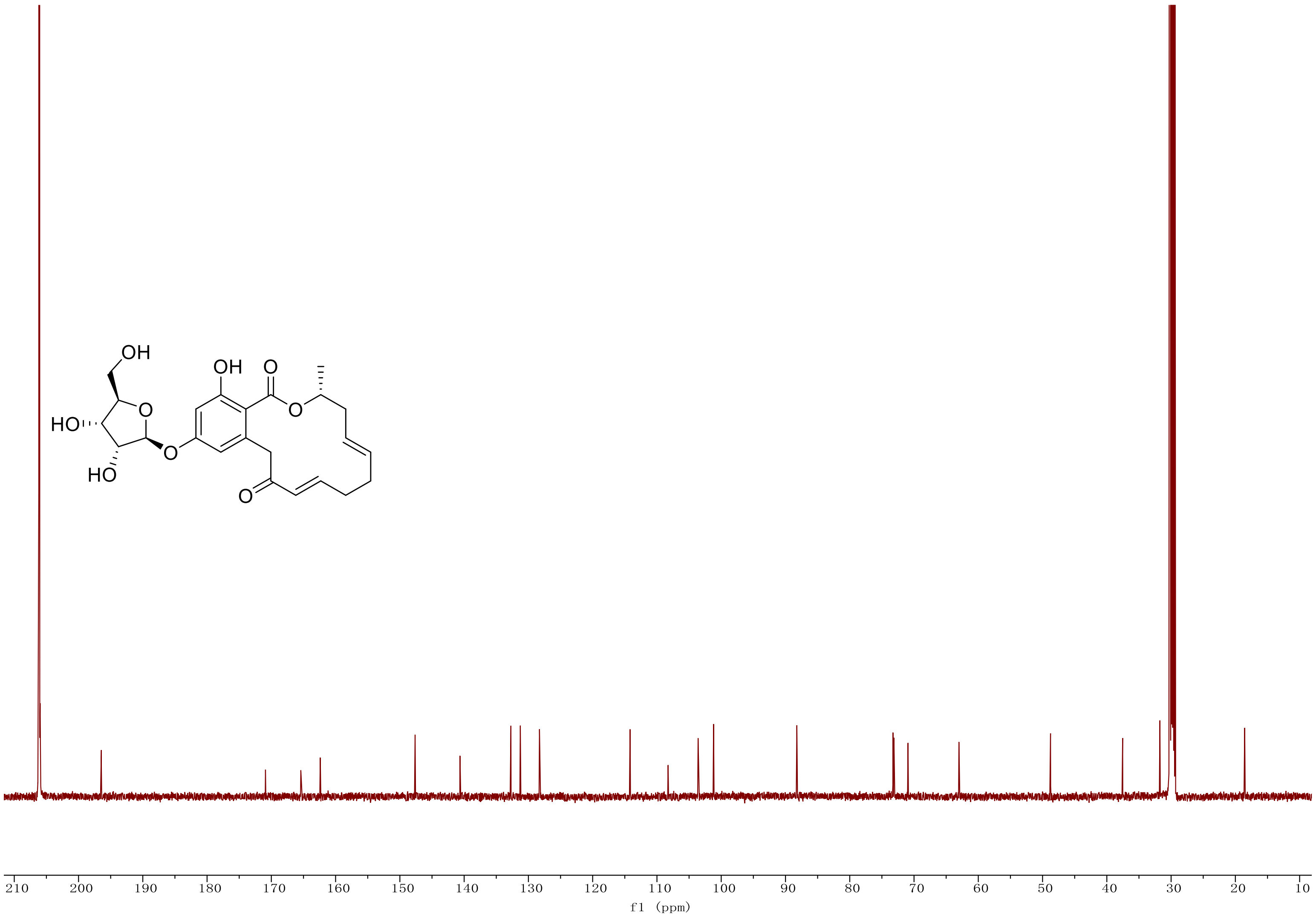

b. ^{13}C NMR spectrum of monocillin II glycoside (**3**; 125MHz, Acetone-d_6)

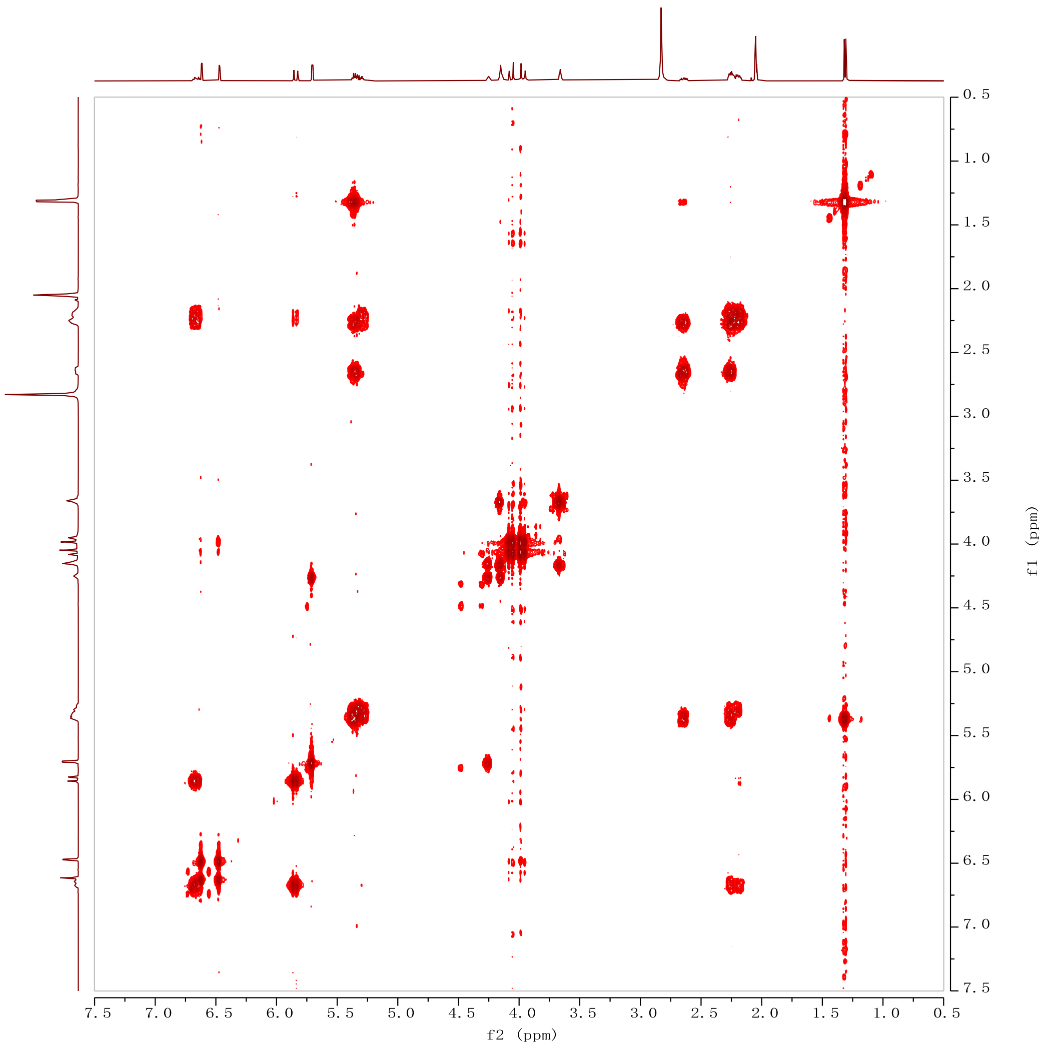

c. The 2D NMR ^{1}H–^{1}H COSY of monocillin II glycoside (**3**)

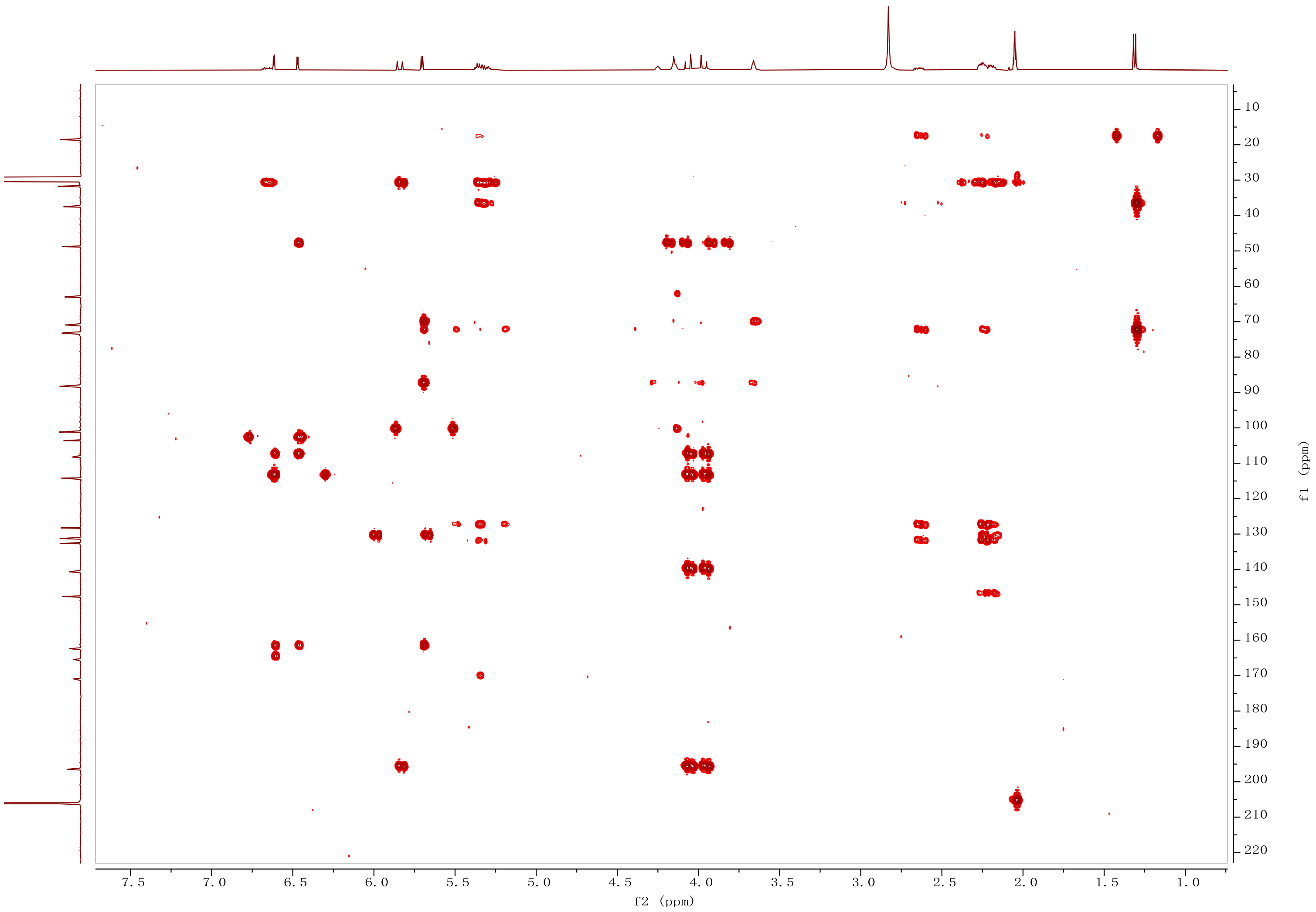

d. The 2D NMR HMBC of monocillin II glycoside (**3**)

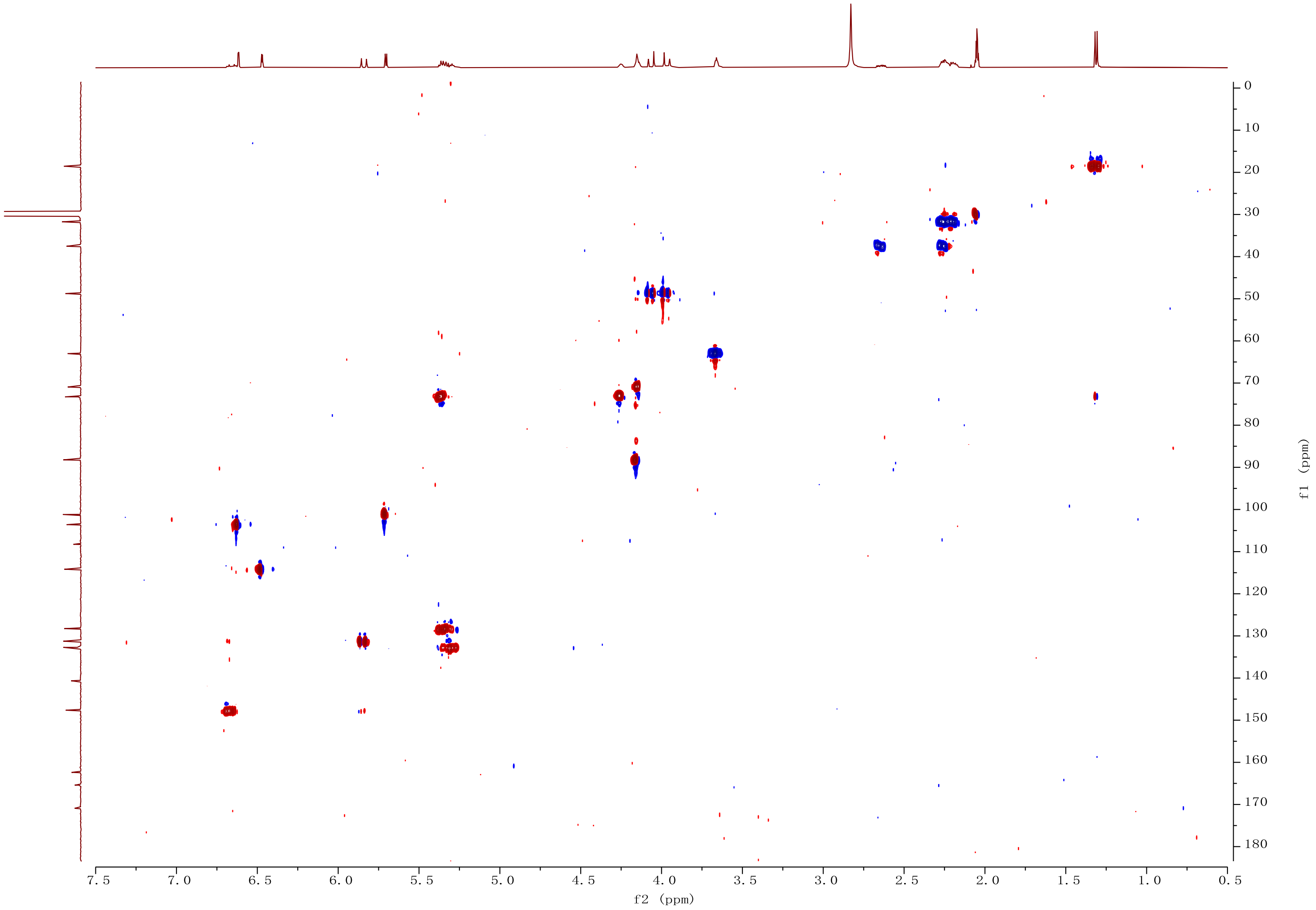

e. The 2D NMR HSQC of monocillin II glycoside (**3**)

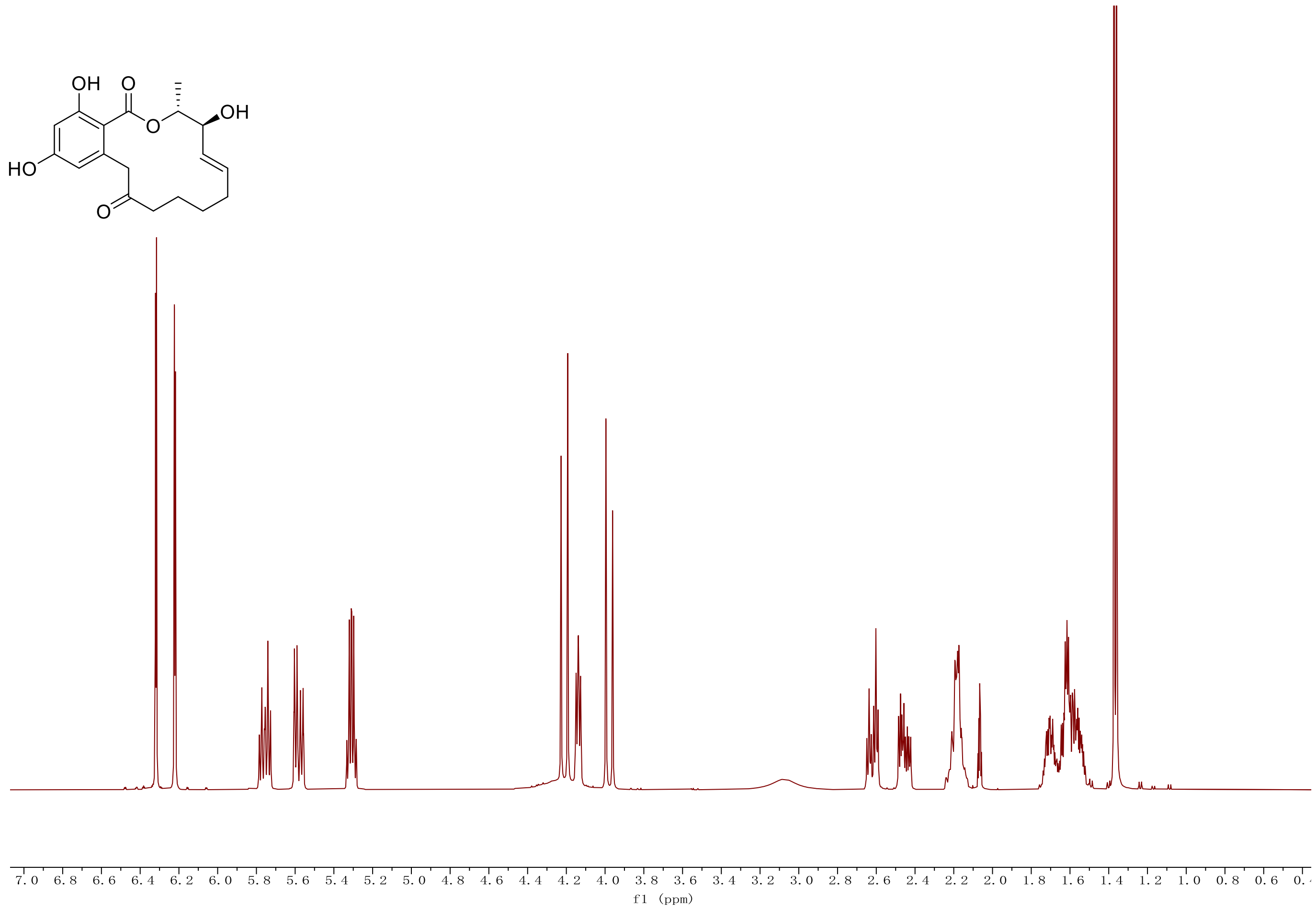

Figure S4**. NMR spectra of monocillin VI (4)**

a. ^{1}H NMR spectrum of monocillin VI (**4**; 500MHz, Acetone-d_6)

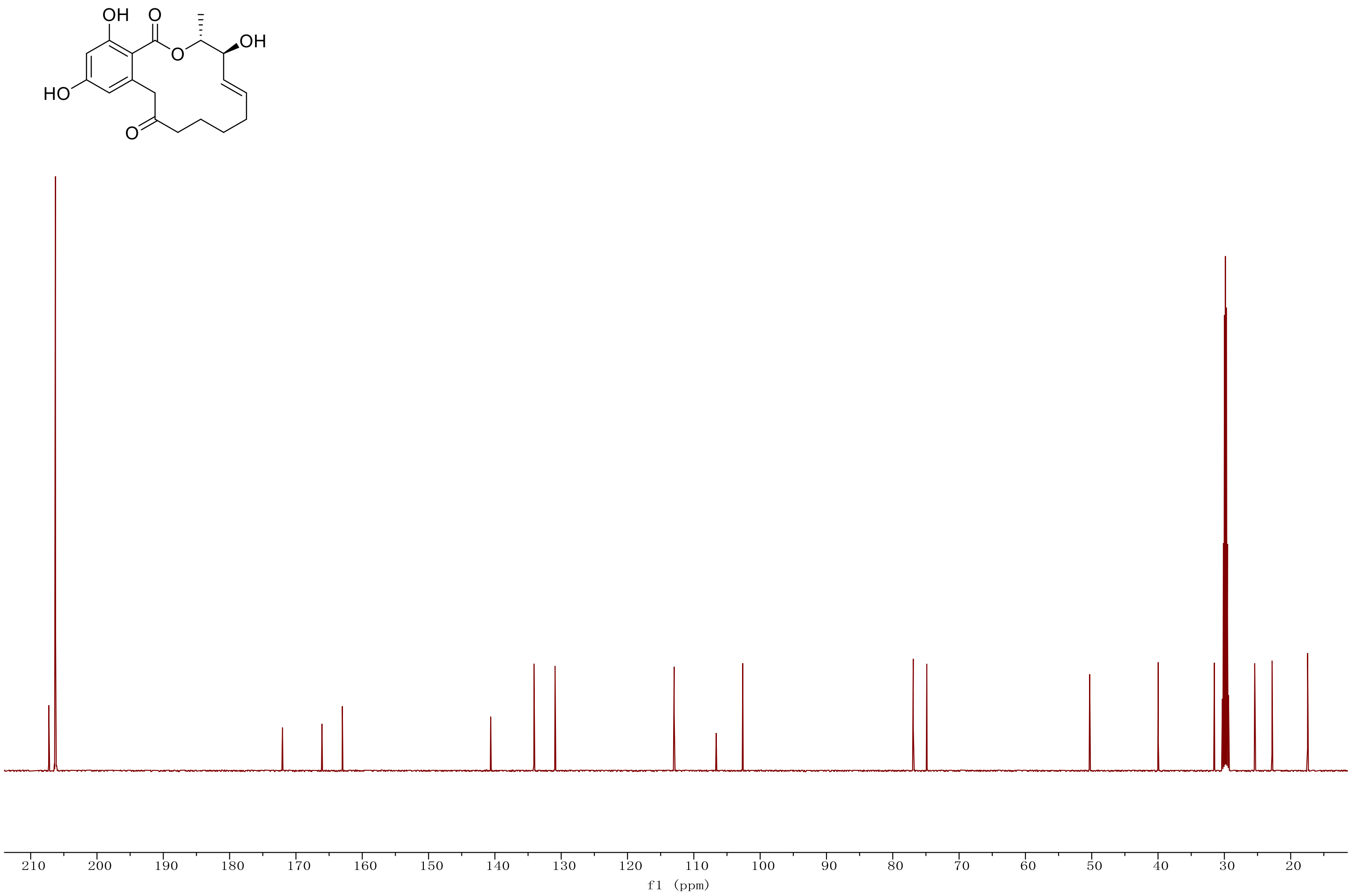

b. ^{13}C NMR spectrum of monocillin VI (**4**; 125MHz, Acetone-d_6)

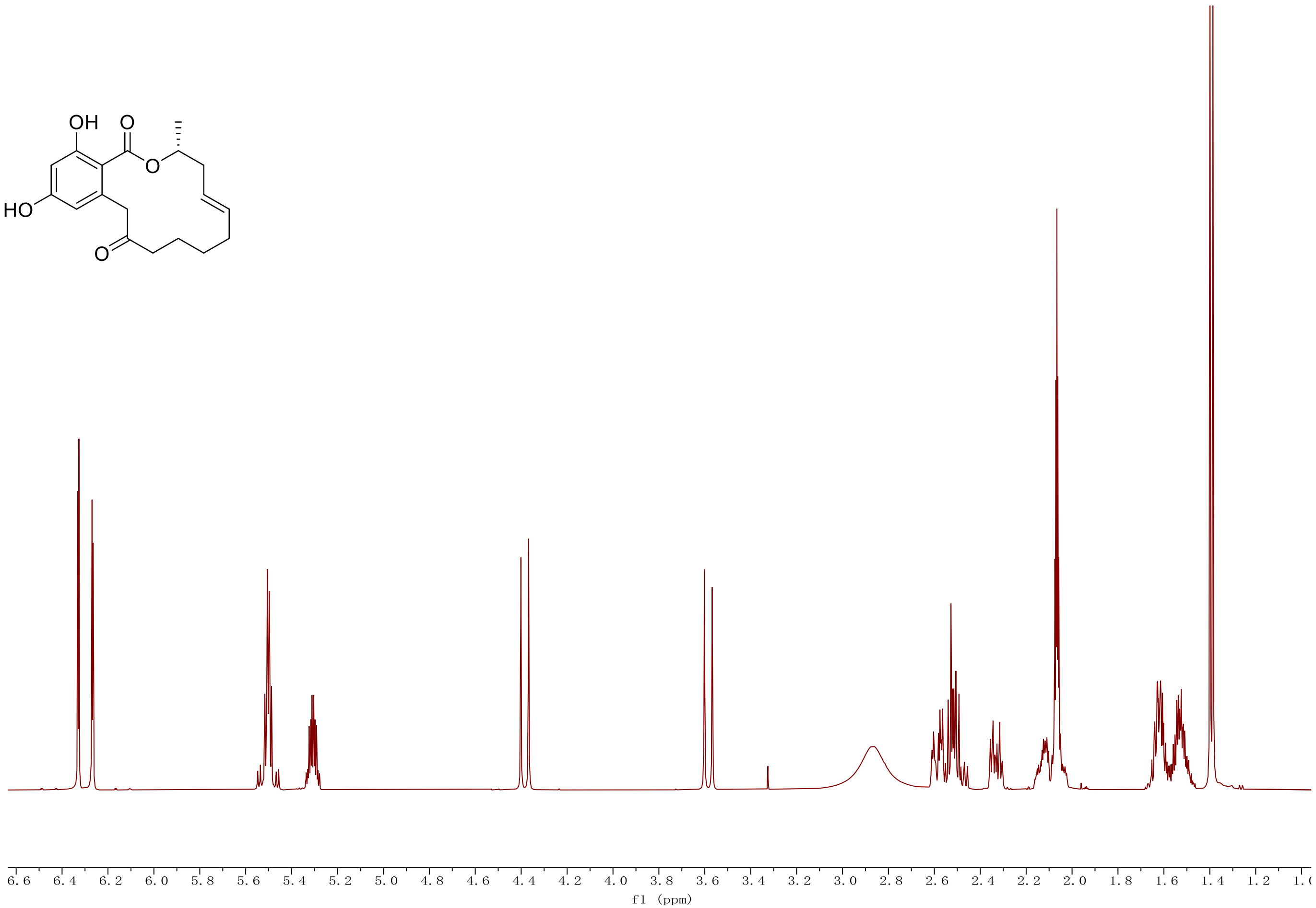

Figure S5. NMR spectra of monocillin IV (5)

a. ^{1}H NMR spectrum of monocillin IV (**5**; 500MHz, Acetone-d_6)

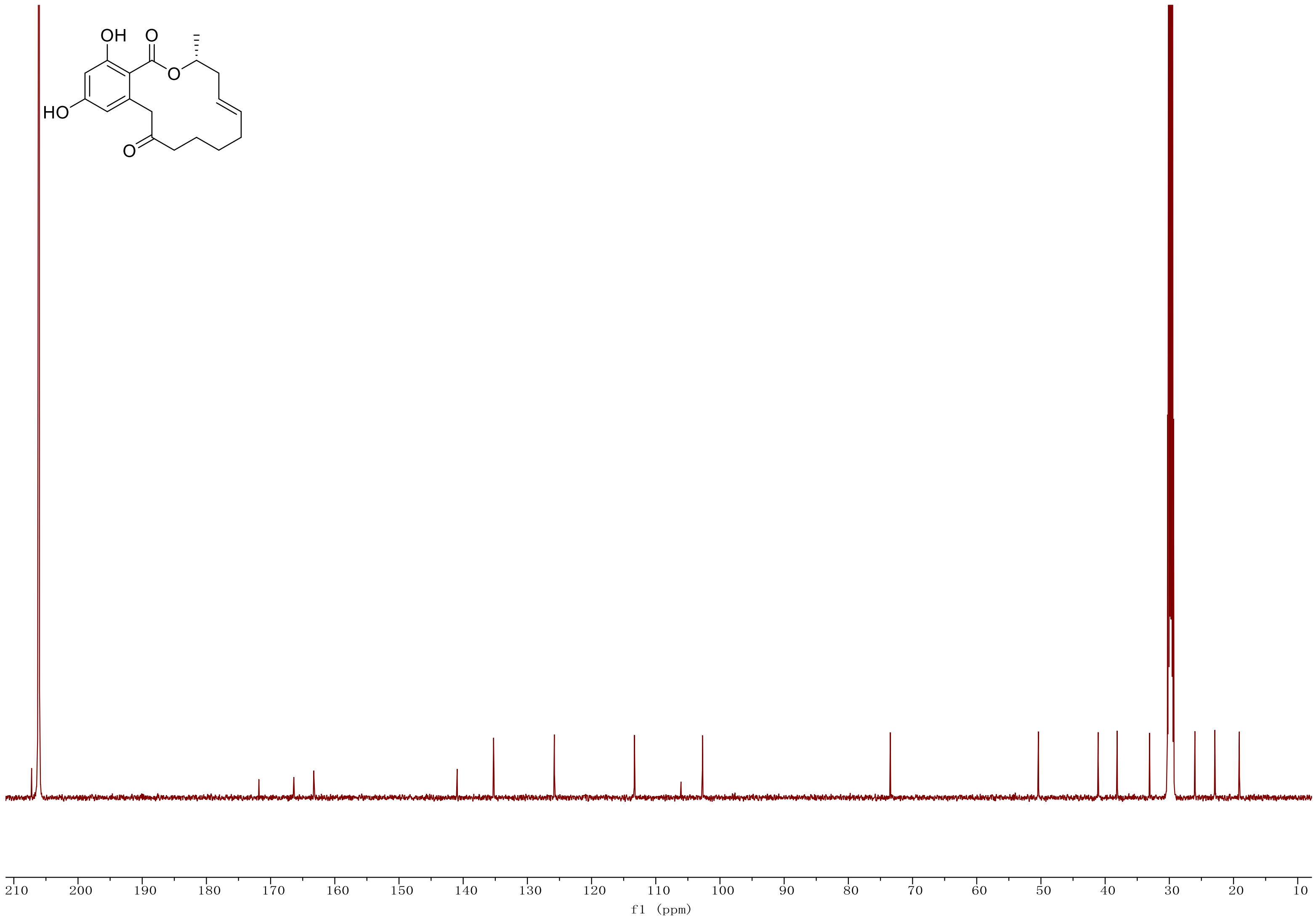

b. ^{13}C NMR spectrum of monocillin IV (**5**; 125MHz, Acetone-d_6)

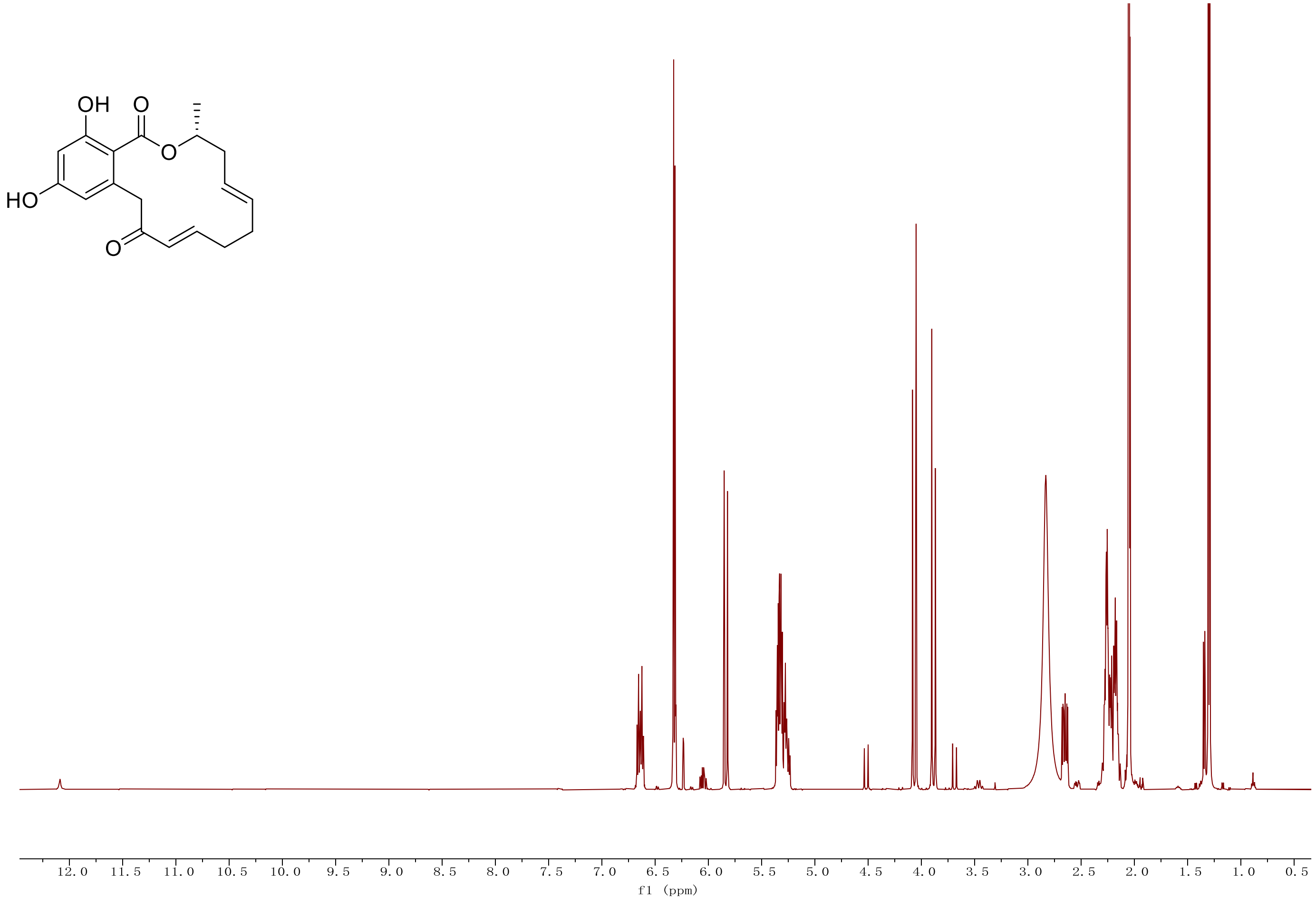

Figure S6. NMR spectra of monocillin II (6)

a. ^{1}H NMR spectrum of monocillin II (**6**; 500MHz, Acetone-d_6)

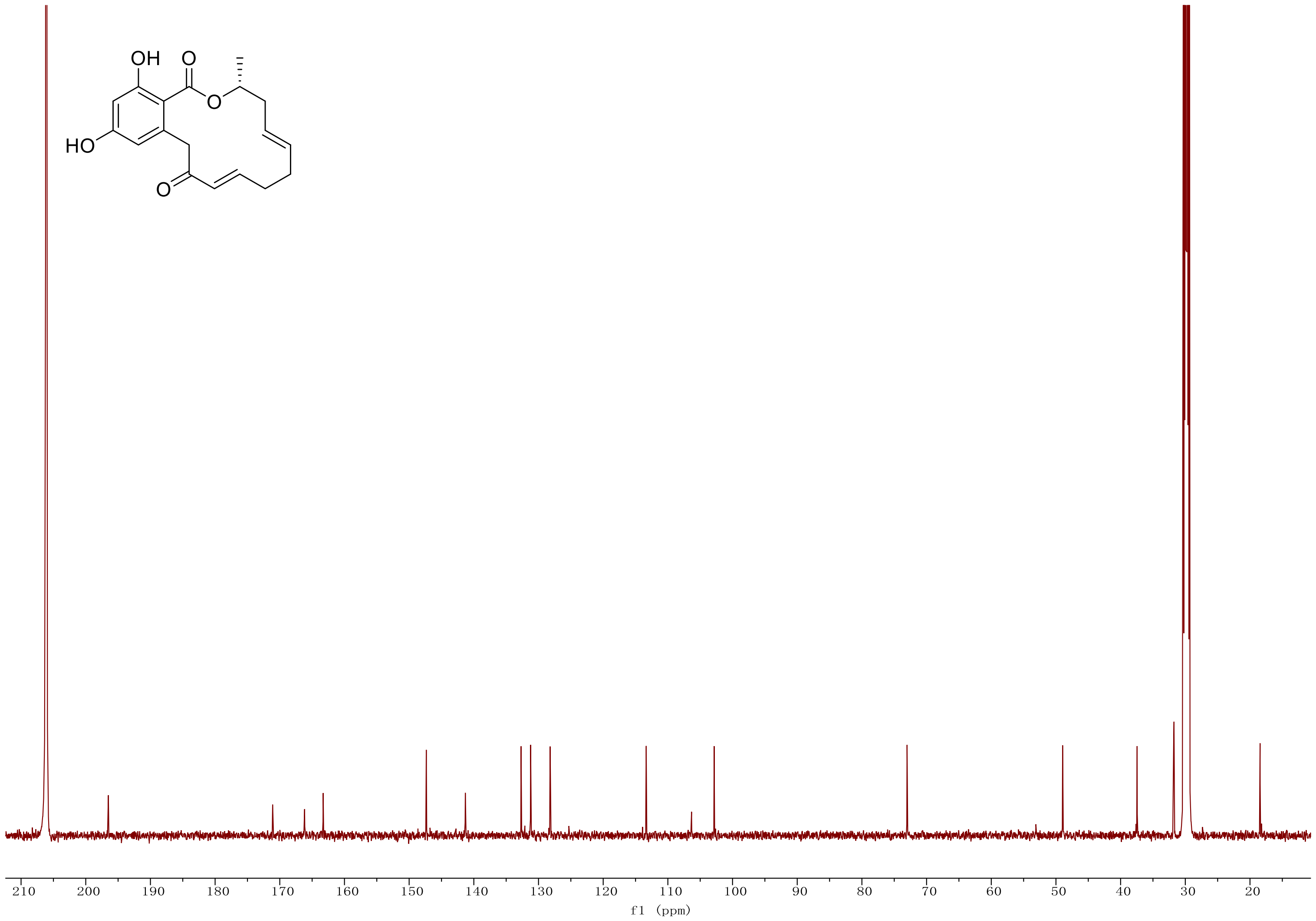

b. ^{13}C NMR spectrum of monocillin II (**6**; 125MHz, Acetone-d_6)

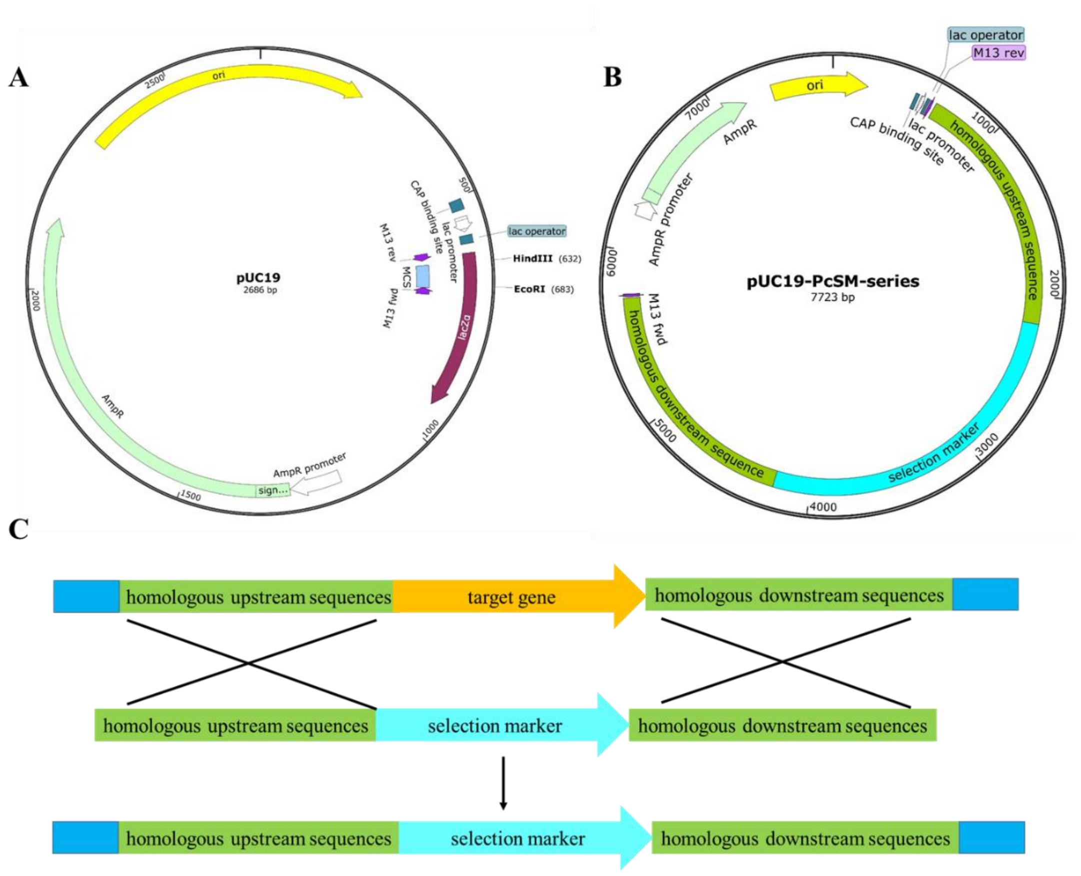

Figure S7. Homologous recombination principle and knockout vector plasmid map.

A. Plasmid map of the vector pUC19 for construction of knockout vectors.

B. Plasmid map of the vector pUC19-PcSM series of knockout vectors.

C. The principle of the gene knockout.

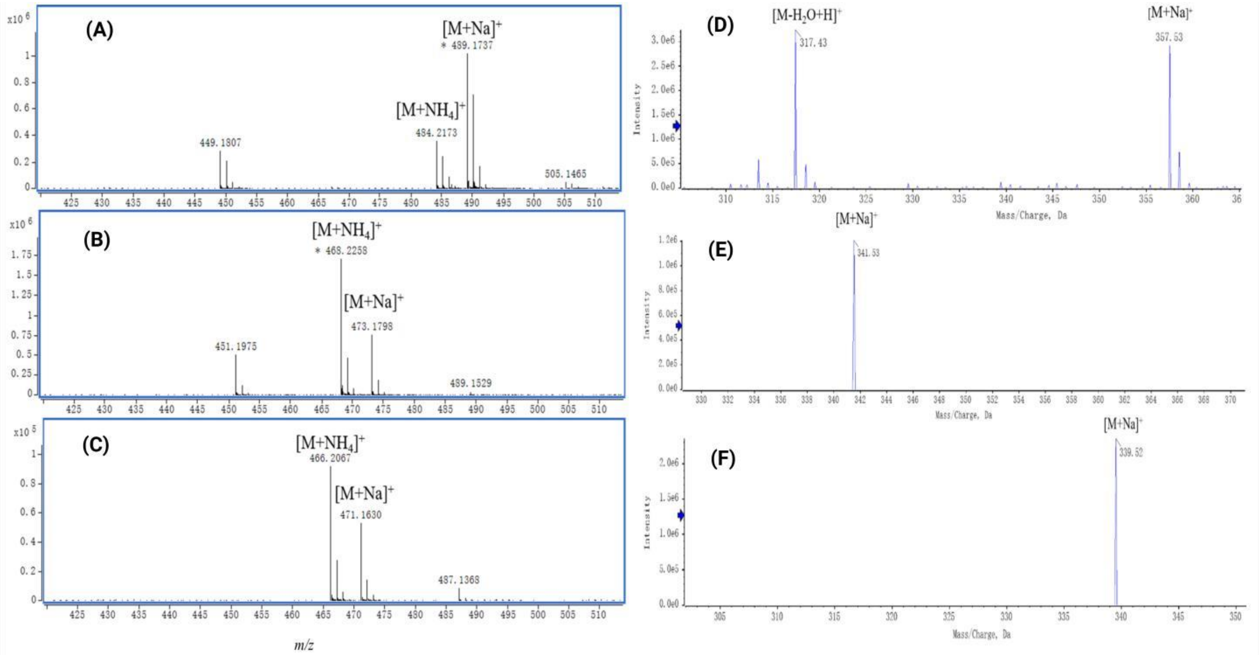

Figure S8. MS spectra of compounds 1-6.

(A-C) HRESI mass spectrum of **(A)** monocillin VI glycoside (**1**), **(B)** colletogloeolactone A (**2**), **(C)** monocillin II glycoside (**3**). **(D-F)** ESI mass spectrum of **(D)** monocillin VI (**4**), **(E)** monocillin IV (**5**), **(F)** monocillin II (**6**).

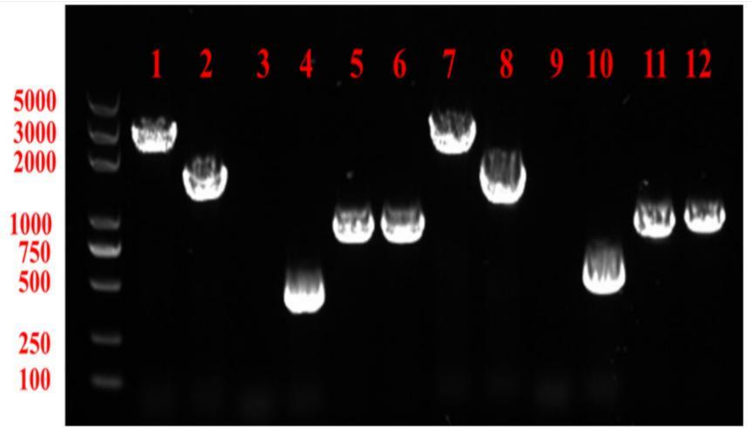

Figure S9. Demonstration of *pchI* and *pchE* genes disruption by PCR.

Lanes 1, 3 and 5: Δ*pchI*; lanes 7, 9 and 11: Δ*pchE*; lanes 2, 4, 6, 8, 10 and 12: wild-type of *Pochonia chlamydosporia* PC-170. Lanes 1 and 2 show PCR products amplified with primer pair Check08959-F and Check08959-R. Lanes 3 and 4 show PCR products amplified with primer pair K08959-F and K08959-R. Lanes 7 and 8 show PCR products amplified with primer pair Check08964-F and Check08964-R. Lanes 9 and 10 show PCR products amplified with primer pair K08964-F and K08964-R. Lanes 5, 6, 11 and 12 show PCR products amplified with primer pair K12324-F and K12324-R.

Table S1a. Primers used for gene knockout.

Primer name	Primer sequence(5'-3')	PCR product (Size in bp)
08959UP-F<br>08959UP-R	aaacgacggccagtgaattcGGCCAGAAATCCCATCTTGC<br>gcgttaatctagaCCTGGTGATCGCCTCAAAGG	pUC19-PcSM08959-UP (1500 bp)
08959NEO-F<br>08959NEO-R	caccaggTCTAGATTAACGCTTACAATTTCCATT<br>cgatgtagtgcctTCTAGAGAATAGGAACTTCGGAATAGG	pUC19-PcSM08959-NEO (2059bp)
08959DOWN-F<br>08959DOWN-R	ctctagaAGGCACTACATCGGACAATCG<br>accatgattacgccaagcttGACCAATTTCGATGAGCATATCG	pUC19-PcSM08959-DOWN (1500 bp)
Check08959-F<br>Check08959-R	tagtcattcgcgccaggtag<br>catccgtctggtctgacaag	Fragment containing the NEO gene (2588 bp)
K08959-F<br>K08959-R	gaagaagcgttctaccatcc<br>cctcatagtcaagtccgttcg	Partial fragment on the *pchI* gene (787 bp)
08964UP-F<br>08964UP-R	aaacgacggccagtgaattcCAACGACGGAAGCATACTCTCG<br>gcgttaatctagaTGGATTCTCTGGATTCTTCAAAGTT	pUC19-PcSM08964-UP (1500 bp)
08964NEO-F<br>08964NEO-R	gaatccaTCTAGATTAACGCTTACAATTTCCATT<br>gaccgtggctagtattgtTCTAGAGAATAGGAACTTCGGAATAGG	pUC19-PcSM08964-NEO (2059 bp)
08964DOWN-F<br>08964DOWN-R	gaACAATACTAGCCACGGTCCTTTCG<br>accatgattacgccaagcttACATAGTTACAACATGACTAGAACGAATG	pUC19-PcSM08964-DOWN (1500 bp)
Check 08964-F<br>Check08964-R	aatgctgaagaccaccaacc<br>aactcgtctatatcgaagcgg	Fragment containing the NEO gene (2579 bp)
K08964-F<br>K08964-R	ctgcacttgacaacgcattac<br>ccacagcctctggaatgtc	Partial fragment on the *pchE* gene (780 bp)
K12324-F<br>K12324-R	CGGATACTTGTTCTCGTTACCG<br>GTCGCACTTCTGTATTGGAGAT	Partial fragment on the *VFPPC_12324* gene (655 bp)
pUC19-F<br>pUC19-R	AAGCTTGGCGTAATCATGGTCA<br>GAATTCACTGGCCGTCGTTTT	Linearized fragments of pUC19 vector (2642 bp)

Table S1b. Vectors and strains used for gene knockout.

Strain and plasmid name	Description	Source
Pochonia chlamydosporia PC-170	*Pochonia chlamydosporia* PC-170 wild-type	Laboratory storage
pUC-Cas9-neo	Amplification selection marker	Laboratory storage
Escherichia coli DH5α strain	plasmid transformation	Tsingke Biotech Co., Ltd.
pUC19	Knockout vector, AMP^r	Tsingke Biotech Co., Ltd.
pUC19-PcSM08959	For knockout of gene *pchI* (*VFPPC_08959*)	This experiment constructs
pUC19-PcSM08964	For knockout of gene *pchE* (*VFPPC_08964*)	This experiment constructs

Table S2. Nematicidal activity of the compounds 1-3.

Treatment	Concentration (μg/mL)	Corrected mortality±SD
**1**	500	91.1±0.5bc
	350	89.8±0.8bc
	250	76.0±2.0d
	125	64.9±1.7e
	62.5	33.7±0.5i
**2**	500	88.4±1.0c
	350	68.6±1.1e
	250	59.5±1.0f
	125	46.0±1.1g
	62.5	27.3±0.8j
**3**	500	96.2±0.5a
	350	93.9±0.9ab
	250	91.2±1.2bc
	125	80.4±5.5d
	62.5	40.0±0.5h
Abamectin	50	93.3±0.4ab

The regression curves for the compounds (**1**-**3**) are as follows: $y = 0.6335x - 0.7504$ $R^2 = 0.9491$, $y = 0.6265x - 0.8658$ $R^2 = 0.9656$, $y = 0.5954x - 0.5706$ $R^2 = 0.8425$. Means that do not share a letter are significantly different ($p < 0.05$).
